# Prognostic significance and immunological role of HPRT1 in human cancers

**DOI:** 10.17305/bb.2023.9775

**Published:** 2024-04-01

**Authors:** Yiwen Lu, Ruixue Chen, Han Zhang, Xu Sun, Xiangjun Li, Mengyuan Yang, Xudong Zhang

**Affiliations:** 1 Department of Oral and Maxillofacial Surgery, Hebei Key Laboratory of Stomatology, Hebei Clinical Research Center for Oral Diseases, School and Hospital of Stomatology, Hebei Medical University, Shijiazhuang, China; 2 Department of Pathology, Shijiazhuang Great Wall Hospital of Integrated Traditional Chinese and Western Medicine, Shijiazhuang, China

**Keywords:** Pan-cancer, hypoxanthine phosphoribosyl transferase 1 (HPRT1), immunity, cancer risk factors, prognosis

## Abstract

Hypoxanthine phosphoribosyl transferase 1 (*HPRT1*), once considered a housekeeping gene, has been identified as playing an important role in several tumors. Its role in pan-cancer, however, has not been systematically studied. This study evaluates the relationship between *HPRT1* and clinical parameters, survival prognosis, and tumor immunity based on multiomics data from The Cancer Genome Atlas (TCGA) and the Gene Expression Omnibus (GEO) databases. Drug sensitivity analysis screened 16 effective drugs against *HPRT1*, exploring the interactions with chemicals and genes. The significance of *HPRT1* in tumor immunotherapy has also been investigated. Immunohistochemistry confirmed significant differences in the expression of HPRT1 between five tumor types (colon adenocarcinoma [COAD], head–neck squamous cell carcinoma [HNSC], lung adenocarcinoma [LUAD], thyroid carcinoma [THCA], uterine corpus endometrial carcinoma [UCEC]), and adjacent normal tissues (*P* < 0.05). *HPRT1* competitive endogenous RNA (ceRNA) network was constructed in HNSC. Through cytological experiments, it was verified that *HPRT1* plays a carcinogenic role in HNSC and is associated with tumor cell proliferation, migration, invasion, and apoptosis. In addition, there was a significant positive correlation between *HPRT1* and programmed cell death-1 (PD-1) expression in HNSC (*P* < 0.05). These findings suggest that *HPRT1* may be a potential biomarker for predicting and treating cancer.

## Introduction

Hypoxanthine phosphoribosyl transferase 1 (*HPRT1*) was previously thought to be a housekeeping gene that encodes enzymes that can participate in the cell cycle by regulating the purine and inosine production, essential for cell growth [1]. HPRT1, as a salvage pathway enzyme, transfers phosphoribose from phosphoribosyl diphosphate to hypoxanthine and guanine bases, forming inosine monophosphate and guanine monophosphate, respectively. These compounds are subsequently converted into functional nucleotides for DNA synthesis and repair [2].

The expression level of HPRT1 is abnormally high in central nervous system tissues, four times higher than in other somatic tissues, although the reason for this elevated expression is unknown [3, 4]. It is worth noting that individuals with complete HPRT1 deficiency develop Lesch–Nyhan syndrome, while partial deficiency leads to gout-like symptoms unique to Kelly–Seegmiller syndrome [3, 5]. Patients with severe Lesch–Nyhan syndrome may suffer from motor dysfunction similar to severe cerebral palsy, intellectual disability, and self-harming behaviors [6].

Recent studies have found HPRT1’s upregulation in various cancers and its involvement in tumor development. These cancers include invasive breast carcinoma (BRCA), head and neck squamous cell carcinoma (HNSC), lung adenocarcinoma (LUAD), lung squamous cell carcinoma (LUSC), prostate adenocarcinoma (PRAD), and uterine corpus endometrial carcinoma (UCEC) [7–11]. HPRT1 was expressed at higher levels in endometrial cancer than in normal tissue and was significantly associated with cancer grade. It was also associated with overall survival (OS) and is sensitive to DNA topoisomerase I (Topo I) and mitogen-activated extracellular signal-regulated kinase (MEK) inhibitors [7]. Compared with normal controls, *HPRT1* RNA levels in breast cancer tissues were significantly elevated, particularly in basal cells and triple-negative breast cancer, suggesting its involvement in cancer progression by positively regulating genes associated with cancer pathways [8]. HPRT1 levels were also significantly upregulated in prostate cancer samples. HPRT1 levels are elevated in approximately 47% of patients compared to normal controls, and it has also been validated as a target antigen for selective cell-mediated killing. In addition, p53 has a significant effect on the expression of HPRT1 in and on the surface of cancer cells [9]. HPRT1 is associated with poorer disease-free survival (DFS) and OS in lung cancer patients. It is also able to promote the formation of lung cancer by inducing neutrophil recruitment and DNA damage repair [10]. The expression of *HPRT1* in HNSC tissues was significantly higher than in normal tissues and is correlated with patient age, sex, pathological stage, and histological grade. Interestingly, The Cancer Genome Atlas (TCGA) cohort demonstrated the absence of significant differences in the expression levels of the *HPRT1* gene between patients with HNSC who were human papillomavirus-positive and -negative [11].

Recent years have seen advancements in immune checkpoint blockade therapy (ICBT) using cytotoxic T-lymphocyte-associated protein 4 (CTLA-4) and the programmed cell death-1/programmed cell death protein-1 (PD-1/PD-L1) pathway. These therapies have shown good prospects in a variety of malignancies [12, 13]. CTLA-4 acts as an immune checkpoint receptor. When T-cells are activated, CTLA-4 can downregulate the function of T-cells by upregulating the plasma membrane, which is critical for maintaining normal immune homeostasis. Antibodies can cause antitumor immunity by blocking CTLA-4 [12]. PD-1, as a negative regulator of T-cell activity, can inhibit the activity of T-cell effectors and promote the depletion of T-cells when interacting with its PD-L1. Therefore, blocking the PD-1 pathway can enhance the reactivity of antitumor T-cells and promote tumor immune control [14]. However, some cancer patients have shown unsatisfactory responses to clinical trials using anti-PD-1 and PD-L1 monoclonal antibodies as immune checkpoint inhibitors [15]. Abnormally high PD-L1 expression on antigen-presenting cells in the tumor microenvironment mediates tumor immune escape [16]. The reasons for poor treatment outcomes may be related to the complexity of the tumor immune microenvironment and the impact of immune escape. Therefore, an in-depth study of the immunosuppressive tumor microenvironment is of great significance in improving the effect of pan-cancer immunotherapy.

This study highlights the multifaceted role of HPRT1 in a variety of cancer types, which provides a theoretical basis for its potential as a novel therapeutic strategy.

## Materials and methods

### Data acquisition

Data were retrieved from the University of California, Santa Cruz (UCSC) Xena dataset portal (http://xena.ucsc.edu/), encompassing RNA transcriptome data (fragments per kilobase million values), somatic mutation data for 33 cancers, and clinical characteristics [17]. These data underpinned the analysis of 33 cancers based on TCGA data, including gene expression difference analysis, gene activity, clinical correlation, survival prognosis, tumor microenvironment, immune cell infiltration, tumor mutation burden (TMB), microsatellite instability (MSI), the gene set enrichment analysis (GSEA), and long non-coding RNA (lncRNA) and mRNA transcriptome data sources for constructing HNSC-related competitive endogenous RNA (ceRNA) network maps. In addition, GSEA data analysis requires two gene set files, “c2.cp.kegg.v7.4” and “c5.go.bp.v7.4”, to be obtained from the Molecular Signature Database (MSigDB: http://www.gsea-msigdb.org/gsea/). Correlation analysis of immune genes was performed on the following website: http://cis.hku.hk/TISIDB/.

The Gene Expression Omnibus (GEO: https://www.ncbi.nlm.nih.gov/geo/) database was used to verify the differential expression of pan-cancer found in the TCGA database and to analyze the correlation between *HPRT1* and PD-L1/PD-1 in triple-negative breast cancer (GSE107764).

The National Cancer Institute (NCI)-60 compound activity data and RNA sequence expression profiles were downloaded from the CellMiner (https://discover.nci.nih.gov/cellminer/home.do) database for drug susceptibility correlation analysis.

RNA sequence data for HNSC were downloaded from the TCGA database (https://portal.gdc.cancer.gov/) (normal [*n*] ═ 44; tumor [*t*] ═ 503). HNSC immunoscore data were downloaded via The Cancer Immunome Atlas (TCIA: https://tcia.at/). These resources were used to explore the relationship between *HPRT1* and HNSC immunotherapy. The miRNA data required for the construction of the ceRNA network were also downloaded from the TCGA database.

### Analysis of the expression and activity of *HPRT1* across cancers

Perl scripts were used to write code for ID transformation on the expression data. The R package “limma” was used to extract the *HPRT1* gene expression. The expression differences of *HPRT1* in the tumor and the normal groups were analyzed using the R packages “plyr,” “reshape2,” and “ggpubr,” employing Wilcoxon test statistical analysis. The median value of tumor expression was used as a reference for sorting.

The 100 genes most associated with *HPRT1* expression in 33 cancers were identified as the active gene sets of this gene. After calculating *HPRT1* activity using single-sample GSEA (ssGSEA), the target gene activity was compared between tumor and normal tissue groups. The following R packages were used: “limma” and “GSEABase,” “plyr,” “reshape2,” and “ggpubr.” The Wilcoxon test was used for statistical analysis. The 33 cancers were ranked according to the median value of tumor activity.

### Validation of *HPRT1* differential expression analysis

The dataset downloaded from GEO included both tumor and normal tissues. In the TCGA data analysis, some tumor types showed significantly higher expression of *HPRT1* in tumor groups compared to normal groups. These types of tumors were revalidated using the GEO dataset. Data not related to the tumor studied in the dataset were excluded.

The datasets cover 14 types of cancers: bladder urothelial carcinoma (GSE3167, *n* ═ 9 *t* ═ 41, unpaired *t*-test), cervical cancer (GSE7803, *n* ═ 10 *t* ═ 24, unpaired *t*-test), head and neck cancer (GSE29330, *n* ═ 5 *t* ═ 13, Mann–Whitney test), kidney chromophobe (KICH: GSE11151, *n* ═ 5 *t* ═ 4, Mann–Whitney test), prostate cancer (GSE38241, *n* ═ 21 *t* ═ 18, Mann–Whitney test), gastric cancer (GSE33428, *n* ═ 8 *t* ═ 19, unpaired *t*-test), cervical cancer (GSE63678, *n* ═ 10 *t* ═ 12, unpaired *t*-test), breast cancer (GSE139038, *n* ═ 18 *t* ═ 18, paired *t*-test), colorectal cancer (GSE10950, *n* ═ 24 *t* ═ 24, paired *t*-test), esophageal cancer (GSE161533, *n* ═ 28 *t* ═ 28, Wilcoxon test), LUAD (GSE75037, *n* ═ 83 *t* ═ 83, Wilcoxon test), thyroid cancer (GSE65144, *n* ═ 9 *t* ═ 9, unpaired *t*-test), liver cancer (GSE60502, *n* ═ 18 *t* ═ 18, unpaired *t*-test), liver cancer (GSE113996, *n* ═ 20 *t* ═ 20, unpaired *t*-test) and rectum adenocarcinoma (READ: GSE75970, *n* ═ 4 *t* ═ 4, Wilcoxon test). Among them, the first seven cancer types feature unpaired samples between tumor groups and normal tissues, whereas the last seven are paired. Perl scripts were utilized for annotating data in GEO datasets. R packages “impute” and “limma” were used to correct the expression data. GraphPad Prism software was used to analyze the difference between *HPRT1* expression in the tumor group and the normal group.

### Immunohistochemistry staining of HPRT1

We collected specimens from the Stomatological Hospital of Hebei Medical University from 2023, including 20 cases of surgically removed HNSC and adjacent normal tissues. Additionally, four types of tumor tissues, colon adenocarcinoma (COAD), LUAD, thyroid carcinoma (THCA), UCEC, and adjacent normal tissues were obtained from Shijiazhuang Great Wall General Hospital of Integrated Traditional Chinese and Western Medicine. The collected specimens were immediately processed as per standard histopathological techniques. Subsequently, the steps were carried out according to the requirements of the PV9000 immunohistochemical detection kit (ZSGB-BIO, China). HPRT1 antibody (Abways Technology, China NO. CY7090, 1:100) was incubated at 4 ^∘^C overnight in 3,3′-diaminobenzidine (DAB) solution to achieve color development, and the antibodies were rinsed with distilled water. Hematoxylin counterstaining was performed, and the slides were sealed with neutral gum. Then, the antibodies were observed under a microscope, and photographs were taken.

Analysis of immunohistochemical section images involved randomly selecting each specimen section from ten fields of view using a high-magnification microscope. The readings were performed in a double-blind manner by two authors under the microscope. Microscopic HPRT1 staining intensity scoring standard ranged as follows: 0 points indicate no staining (−), 1 point indicates light staining (+), 2 points indicate moderate staining (++), and 3 points indicate heavy staining (+++). The scoring criteria for the percentage of positive cells were as follows: 0 points for no positive cells, 1 point for positive cells ranging from 1% to 25%, 2 points for positive cells ranging from 26% to 50%, and 3 points for cases where more than 50% of cells were positive. The scores obtained by the above two scoring methods were added and are categorized as a negative group which includes ranges of 0–2 (−) and a positive group which includes a ranges of 3–4 (+) and 5–6 (++). Cytoplasmic staining appearing brownish-yellow or tan is designated as HPRT1-positive.

### Analysis of clinical differences in HPRT1 across cancers

Patients were categorized based on different clinical features (age, sex, stage, survival status), in order to analyze HPRT1 expression variations in the aforementioned groups. In the clinical trait of age, we divided patients into younger than or equal to 65 years old and older than 65 years old. We observed the correlation between HPRT1 expression and age group across various cancer types. Similarly, HPRT1 expression analysis, patients were divided into female and male. The cancer stages of patients were divided into stages I, II, III, and IV, while their status was divided into survival or death. The Wilcoxon test was used for statistical analysis of the data, by utilizing R package “ggpubr.”

### Survival analysis of *HPRT1* across cancers

We investigated the prognostic value of *HPRT1* in various cancer types using univariate Cox regression analysis and Kaplan–Meier survival analysis. Patients were divided into two groups based on *HPRT1* gene expression levels to compare survival differences utilizing the Kaplan–Meier survival analysis. Cox regression analysis was used to compare the expression of the *HPRT1* gene as a continuous variable against the survival time and status. A hazard ratio (HR) > 1 indicates that *HPRT1* is a high-risk gene in the tumor, whereas HR < 1 suggests that *HPRT1* is a low-risk gene. OS, disease-specific survival (DSS), DFS, and progression-free survival (PFS) were selected to investigate the correlation between *HPRT1* expression and prognosis. The R packages used were “limma,” “survival,” “survminer,” and “forestplot.”

### Correlation analysis of *HPRT1* expression and immunity

Tumor microenvironment analysis involved assessing the relationship between *HPRT1* expression or activity and the four scores generated by the ESTIMATE algorithm (StromalScore, ImmuneScore, ESTIMATEScore, and TumorPurity). Tumor purity refers to the proportion of tumor cells in tumor tissue. Regarding the StromalScore or ImmuneScore, the higher the stromal score or immune score, the higher the content of matrix components or immune components. The sum of the two values is the ESTIMATEScore score, with higher scores indicating lower tumor purity. Correlation coefficient filters (cor > 0.3, *P* value < 0.05) and Spearman correlation analysis were applied using “estimate,” “limma,” “ggplot2,” “ggpubr,” and “ggExtra” R packages. Additionally, we explored the association between *HPRT1* expression and tumor microenvironment across various cancers, using the R packages “limma,” “reshape2,” and “ggpubr” in conjunction with the Wilcoxon test.

Immune cell infiltration analysis used CIBERSORT to assess the relative abundance of pan-cancer-infiltrating immune cell subsets [18]. The correlation between *HPRT1* expression and infiltration of 22 immune cells in 33 cases of cancer was calculated using “limma,” “ggplot2,” “ggpubr,” and “ggExtra” R packages for scoring applications. Correlation coefficient filters were as follows: cor > 0.3, *P* value < 0.001. The correlation method utilized is Spearman. Further analysis of the relationship between *HPRT1* expression and immune cells across different cancers was carried out, employing the R packages “estimate,” “limma,” “ggplot2,” “ggpubr,” and “ggExtra.” The method utilized for this part of the study was the Wilcoxon test.

Immunogene correlation analysis examined the relationship of HPRT1 expression to three immunomodulator-related genes, including immunoinhibitors, immunostimulators, and major histocompatibility complex (MHC) molecules, using data from the integrated repository portal for tumor-immune system interactions database (TISIDB). In the corresponding three heatmaps, red indicates that the immune-related gene is positively correlated with *HPRT1*, and blue represents a negative correlation with it. The positive and negative correlation values in the heatmap were collected and a scatterplot of the gene composition with the *HPRT1* gene was also generated.

TMB and MSI analyses assessed the correlation information of pan-cancer *HPRT1* with TMB and MSI, respectively. The Spearman method was used to assess the correlation of *HPRT1* with TMB and MSI. Perl was used to extract TMB values for 33 cancers. The R package “fmsb” was used for correlation analysis.

The GSE107764 dataset (*n* ═ 48) was sourced from the GEO database to verify the therapeutic effect of *HPRT1*. This dataset studies the correlation between triple-negative breast cancer and PD-1/PD-L1 expression. According to the immunohistochemical scoring of the companion paper in this dataset, the expression of PD-1/PD-L1 antibody in tissues increased sequentially from 0 to 5 [19]. We recorded points 0–2 as the low-expression group and 3–5 as the high-expression group. The R packages “limma,” “ggplot2,” and “ggpubr” were used to verify the correlation between *HPRT1* and PD-1/PD-L1 in this dataset and then indirectly verified the relationship between target genes and immunotherapy. The Wilcoxon test was used as a statistical method for this section.

### GSEA enrichment analysis

We utilized GSEA enrichment analysis to understand the biological processes involved in *HPRT1* across 33 cancers. Utilizing the “c2.cp.kegg.v7.4” and “c5.go.bp.v7.4” gene sets from the MSigDB, we could identify the active functions or pathways associated with both high and low *HPRT1* expression groups. Signal pathways with *P* values less than 0.05 were filtered out. The R packages used for this analysis were “limma,” “org.Hs.eg.db,” “clusterProfiler,” and “enrichplot.”

### Drug sensitivity analysis

We performed drug sensitivity analysis on *HPRT1* by downloading NCI-60 compound activity data and RNA sequence expression profiles from the CellMiner database. This analysis focused on drugs approved by the United States Food and Drug Administration (FDA) or clinical trial-approved drugs. The “impute,” “limma,” “ggplot2,” and “ggpubr” R packages were used [20], with Pearson’s method for correlation analysis.

### Interaction of *HPRT1* with chemicals and genes

The public Comparative Toxicogenomics Database (CTD: http://ctdbase.org/) is a website that links toxicological information on chemicals, genes, phenotypes, diseases, and exposures to study how chemicals relate to molecular mechanisms and their overall impact on health [21]. Through this database, we identified chemicals interacting with *HPRT1* and genes highly similar to *HPRT1* in terms of chemical interactions. During the screening process for chemical interactions, references were configured in descending order.

GeneMANIA (http://genemania.org) is a website for generating gene function predictions and prioritizing genes for functional analysis. In addition, the site uses a large amount of genomic and proteomic data (including GEO, IRefIndex, BioGRID, and I2D) to identify genes with functions similar to the gene of interest. It also analyzes interactions between genes or proteins [22, 23]. By examining the function of similar genes in GeneMANIA, we identified genes that function similarly to *HPRT1*.

### Further analyses of *HPRT1* expression in HNSC

#### Immunotherapy analysis of HPRT1 in HNSC

After downloading the HNSC data from the TCGA database, sample data were merged, and ID conversion was performed using Perl. The data were categorized into normal and tumor groups based on *HPRT1* expression levels. The TCGA dataset underwent preprocessing, wherein the normal group data of TCGA was excluded. Subsequently, the remaining dataset was stratified into two groups, characterized by differential expression levels of *HPRT1*: one displaying high expression and the other exhibiting low expression. HNSC immunoscore data were downloaded via TCIA. The aforementioned two sets of data samples were analyzed to assess the effectiveness of immunotherapy by investigating potential differences in CTLA4 and PD-1 expression between the *HPRT1* high-expression group and the low-expression group. The analysis utilized R packages, specifically “limma,” “ggplots,” and “ggpubr” and employed the Wilcoxon test to discern any significant distinctions.

#### Screening of HPRT1-associated lncRNAs and microRNAs in HNSC

We downloaded the miRNA transcript data from HNSC from TCGA, merging the data using Perl scripts. The ID conversion for miRNA was performed using files from miRBase (https://www.mirbase.org/). *HPRT1*-related miRNAs were identified using starBase (https://starbase.sysu.edu.cn/), screening for microRNA targets associated with *HPRT1* across seven databases (PITA, RNA22, miRmap, microT, miRannda, PicTar, TargetScan). In two or more databases, microRNAs associated with *HPRT1* were screened, and the Cytoscape software was used to construct *HPRT1*-related miRNA–mRNA regulatory network diagrams.

Correlation analysis was performed on *HPRT1* and miRNAs, and the screened miRNAs met the criteria of cor < −0.1, *P* < 0.001. The miRNAs identified in the previous step were assessed for differential expression between tumor and normal tissues. Those miRNAs exhibiting low expression in tumor tissues and having a significance value of *P* < 0.05 were selected. The R packages used were “limma,” “reshape2,” “ggpubr,” and “ggExtra”. The relationship between miRNA and survival prognosis was conducted through Kaplan–Meier survival analysis. Selected were miRNAs with a significance value of *P* < 0.001. Additional R packages, specifically “limma,” “survival,” and “survminer” were employed for the analysis, utilizing the Spearman and Wilcoxon tests as methods.

The screened miRNAs were further analyzed to identify the lncRNAs associated with them. The lncRNAs linked with the target miRNA were sourced from the starBase database. The correlation between these lncRNAs and the target miRNAs was analyzed, specifically focusing on lncRNAs that showed a significant negative correlation with miRNAs and met the screened conditions as follows: cor < −0.1, *P* < 0.001. Cytoscape software was used to construct a single-gene ceRNA network diagram. Differential expression analysis was performed on the identified lncRNAs in tumor and normal tissues, and significantly different lncRNAs (*P* < 0.05) were selected. Additionally, *HPRT1* and screened lncRNAs were analyzed for correlation analysis, where lncRNAs (cor > 0.1, *P* < 0.001) positively correlated with *HPRT1*, hence they were selected. The R packages used were “limma,” “reshape2,” “ggpubr,” and “ggExtra”. Finally, the Kaplan–Meier survival analysis method was applied to screen lncRNAs related to survival prognosis (*P* < 0.05), using the R packages “limma,” “survival,” and “survminer.” Statistical methods used were the Spearman and Wilcoxon tests.

### RNA isolation and quantitative real-time reverse transcription PCR

RNA was extracted using Trizol. Applied Biosystems (ABI) real-time fluorescence quantitative PCR Q5 (Thermo Fisher, MA, USA) was used for carrying out the quantitative reverse transcriptase polymerase chain reaction (RT-qPCR). *GAPDH* is used as an internal housekeeping gene. The specific primers used in this study are shown in Table 1.

**Table 1:** The primers for individual genes

**Primer name**	**Sequence (5’-3’)**
HPRT1(F)	ATTCTTTGCTGACCTGCTGGATTAC
HPRT1(R)	ACTTTTATGTCCCCTGTTGACTGGT
PD-1(F)	ACCCTGGTGGTTGGTGTCGTG
PD-1(R)	TGGCTCCTATTGTCCCTCGTGC
GAPDH(F)	GAAGGTGAAGGTCGGAGTC
GAPDH(R)	GAAGATGGTGATGGGATTTC

HPRT1: Hypoxanthine phosphoribosyl transferase 1; PD-1: Programmed cell death-1.

### Western blotting

Total protein was harvested from the sample buffer. According to the manufacturer’s instructions, protein concentration was quantified using the bio-barcode assay (BCA) method detection kit. The same amount of protein in each sample is separated by electrophoresis on sodium dodecyl sulfate/polyacrylamide gel and then transferred to the polyvinylidene fluoride (PVDF) membrane. Incubate PVDF with primary antibody HPRT1 (Abways Technology, China NO. CY7090, 1:1000) and PD-1 (Proteintech, China NO. 66220-1-lg, 1:5000) at 4 ^∘^C overnight. GAPDH is used as an internal reference protein.

### Cell transfection

Cells in the logarithmic growth phase were seeded with 5 × 10^5^ cells per well on a 6-well plate. After overnight cultivation, transfection is carried out when the cell density reaches 70% the next day. Solution A: Use 125-µL Opti-MEM (Gibco, CA, USA) diluted siRNA (100 pmol/well); Liquid B: Use 125-µL Opti-MEM Dilution Lipofectamine™ 3000 (Thermo Fisher, MA, USA) (5 µL/well). Solutions A and B were gently mixed, allowed to stand for 5 min, combined in a 1:1 ratio, mixed well, and left at room temperature for 15 min before being added to the corresponding group of 6-well plates. The cells were then incubated at 37 ^∘^C in a cell culture incubator for 48 h. After successful transfection, small interfering non-coding RNA (si-NC and si-HPRT1) and si-HPRT1 were obtained.

### 5-Ethyl-2’-deoxyridine (EdU) cell proliferation experiment

The corresponding group of cells (si-NC and si-HPRT1) were digested and collected separately, and 10^5^ cells were taken from each well to prepare cell slides in a 24-well plate, which were then cultured overnight. After staining with EdU staining kit (BeyoClick™ EdU Cell Proliferation Kit with Alexa Fluor 594, Biotime, Shanghai, China), images were captured using a fluorescence microscope.

### Cell Counting Kit-8 (CCK-8) cell proliferation experiment

Cells were grouped (si-NC and si-HPRT1) and inoculated with 10^4^ cells per well on a 96 well plate, respectively. After 24 hours, both groups of cells were subjected to CCK8 (Zeta LIFE, CA, USA) detection.

### Wound-healing

Cells were divided into groups (si-NC and si-HPRT1) for planking, and 10^6^ cells were inoculated into a lined 6-well plate for overnight cultivation. The next day, the 10-µL gun head was used to draw two parallel lines in the vertical direction of the marking line. Serum-free culture medium was used to culture cells, and the images were captured time-dependently ranging from 0 to 24 h.

### Transwell cell invasion experiment

On the ice, the Matrigel gel was diluted with serum-free cell culture medium to 300 µL/mL, and 100 µL was evenly applied to the surface of the cell membrane. Then, the cells were placed in a 24-well plate at 37 ^∘^C for about 3 h. What is then added to the lower chamber is the 600 µL of culture medium containing 10% serum. After cell grouping (si-NC and si-HPRT1), 100-µL of cell suspension is seeded in the upper chamber of each well, for a total of 1 × 10^4^ cells. After 24 h, cells were fixed with 4% paraformaldehyde, air dried in the chamber, and stained with 0.1% crystal violet. Then the crystal violet was rinsed on the surface with tap water and the upper layer was gently wiped off of non-migrated cells. Photos of the non-cellular side were captured under a microscope.

### Annexin V-fluorescein isothiocyanate (FITC)/propidium iodide (PI)-staining assay

Flow cytometry assay kit was used to analyze the cell apoptosis rate according to the standard manufacturer’s protocol (No. C1062S, Biotime, Shanghai, China). The cells were digested by trypsin without EDTA, washed in PBS, and centrifuged at a cell density of 1 × 10^6^ or more per milliliter. After adding Annexin V-FITC and PI, the mixture was incubated at room temperature in darkness for 20 min. Flow cytometry analysis was performed after staining, and the cell apoptosis rate was calculated based on the proportion of Annexin V-positive cells. Normal cells are designated as Q2-3, Q2-4 as early apoptotic cells, Q2-2 as cells in the late stage of apoptosis, and Q2-1 as necrotic cells. The horizontal apoptosis axis (FITC-A) represents Annexin V FITC positive cells, while the vertical apoptosis axis (PI-A) represents PI positive cells.

### Ethical statement

All datasets in the present study were obtained from public databases, including TCGA, GEO, and the TCIA database. These public databases allowed researchers to download and analyze public datasets for scientific purposes. The human tissue used in this experiment was reviewed by the Ethics Committee of Stomatological Hospital of Hebei Medical University (NO. [2023] 002) and the Ethics Committee of Shijiazhuang Great Wall Hospital of Integrated Traditional Chinese and Western Medicine (NO. [2023] 013).

### Statistical analysis

All data on *HPRT1* gene expression levels were normalized using log2 transformation. The correlation analysis in the study was based on Spearman’s and Pearson’s rank correlation coefficients. The median was used to divide patients into both high and low groups. In the Kaplan–Meier survival analysis, specifically, the log-rank test was used to determine the statistical significance. Statistical analysis of the data in this paper was performed by R software (version 4.2.1) and GraphPad Prism (version 9.3.0). Strawberry Perl (version 5.30.0.1) facilitated data merging and ID conversions. Cytoscape software (version 3.9.1) was employed to build miRNA–mRNA regulatory network diagrams and ceRNA network graphs. Differences between the two groups were analyzed using *t*-tests and paired *t*-tests. Nonparametric data were assessed using the Mann–Whitney test and the Wilcoxon test. Significance levels are indicated as follows: * for *P* < 0.05; ** for *P* < 0.01; *** for *P* < 0.001.

## Results

### Differential expression of *HPRT1*

**Figure 1.**
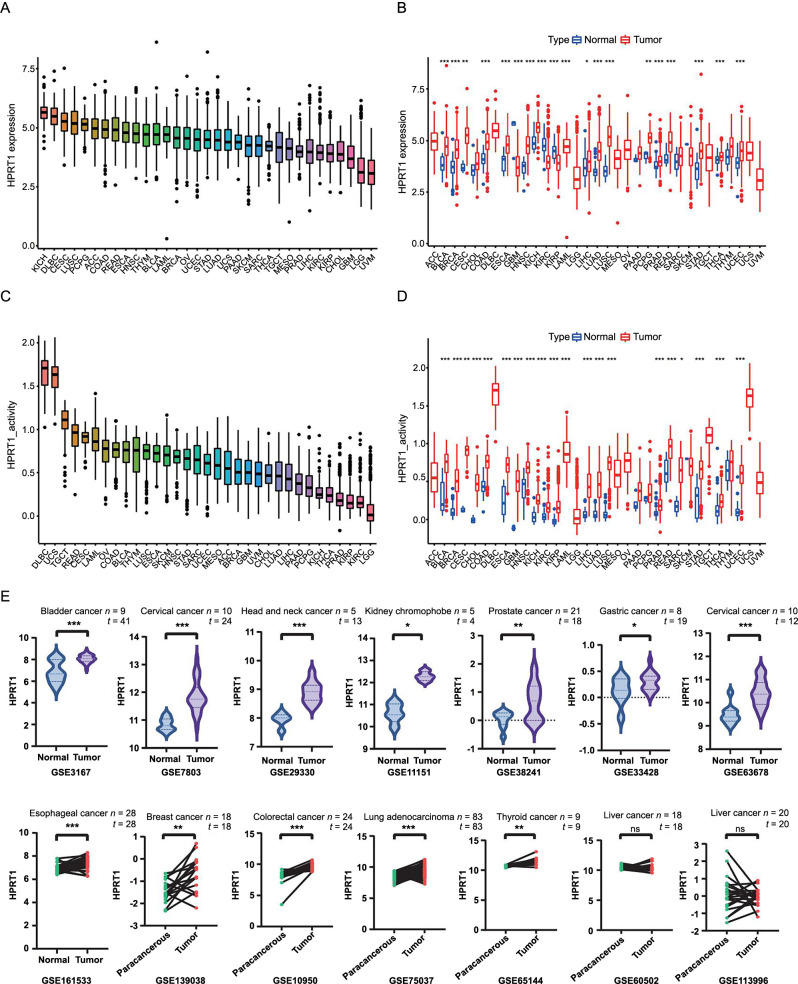
(A) *HPRT1* expression levels in 33 tumors were ranked from high to low; (B) In pan-cancer, there were differences observed in *HPRT1* expression and normal samples; (C) *HPRT1* activity was ranked across 33 tumors, with decreasing activity from left to right; (D) In pan-cancer, *HPRT1* activity differed between normal and tumor groups; (E) The GEO database validated the differential expression of *HPRT1* in a variety of tumors and corresponding normal or paracancerous tissues. *P < 0.05; **P < 0.01; ***P < 0.001. HPRT1: Hypoxanthine phosphoribosyl transferase 1; GEO: Gene Expression Omnibus; ns: Not significant.

The expression of *HPRT1* was different in various tumor tissues (Figure 1A). Based on the TCGA data, *HPRT1* was differentially expressed in 19 cancer tissues compared to corresponding normal tissues. *HPRT1* was upregulated in tumor tissues of bladder urothelial carcinoma (BLCA), BRCA, endocervical adenocarcinoma (CESC), COAD, esophageal carcinoma (ESCA), HNSC, KICH, liver hepatocellular carcinoma (LIHC), LUAD, LUSC, pheochromocytoma, and paraganglioma (PCPG), PRAD, READ, stomach adenocarcinoma (STAD), THCA, and UCEC. Conversely, *HPRT1* was upregulated in normal tissues of glioblastoma multiforme (GBM), kidney renal clear cell carcinoma (KIRC), and kidney renal papillary cell carcinoma (KIRP) (Figure 1B).

The activity of *HPRT1* also varied across different tumors (Figure 1C). *HPRT1* activity was higher in the tumor groups of BLCA, BRCA, CESC, cholangiocarcinoma (CHOL), COAD, ESCA, GBM, HNSC, KICH, KIRC, KIRP, LIHC, LUAD, LUSC, PRAD, READ, sarcoma (SARC), STAD, THCA, and UCEC (Figure 1D).

Since the normal groups of adrenocortical carcinomas (ACC), diffuse large B-cell lymphoma (DLBC), acute myeloid leukemia (LAML), lower-grade glioma (LGG), mesothelioma (MESO), ovarian cancer (OV), testicular germ cell tumors (TGCT), uterine carcinosarcoma (UCS), and uveal melanoma (UVM) are missing in the TCGA database, whether there are differences in *HPRT1* expression and activity in these tumors needs to be further explored.

### GEO dataset validation analysis of HPRT1

Analysis of 14 different tumors, using the GEO dataset yielded results consistent with TCGA database findings. However, no significant difference in HPRT1 expression was observed in liver cancer compared to adjacent tissues. The HPRT1 content in tumor tissue from the READ dataset was higher than in corresponding paracancerous tissue. However, statistical analysis was not feasible due to the small sample size (Figure 1E).

### Immunohistochemical staining of HPRT1

**Figure 2.**
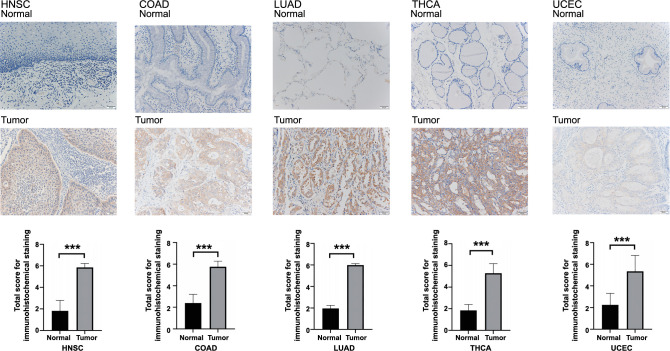
**HPRT1 showed strong positive expression in five cancers, while in corresponding normal tissues, it exhibited either weak positive expression or was not expressed.** Both findings are statistically significant (***P < 0.001). HPRT1: Hypoxanthine phosphoribosyl transferase 1. HNSC: Head-neck squamous cell carcinoma; COAD: Colon adenocarcinoma; LUAD: Lung adenocarcinoma; THCA: Thyroid carcinoma; UCEC: Uterine corpus endometrial carcinoma.

Our experiment also confirmed HPRT1’s positive signal transduction localized in the cytoplasm and cell membrane. It showed strong positivity in HNSC tissues, and either negative or weak positivity in adjacent normal tissues. This pattern was also observed in COAD, LUAD, THCA, and UCEC tumors and corresponding normal tissues. Interestingly, in one of the cases, UCEC showed partial strong positive expression in the corresponding adjacent normal tissue. Its location was in the proliferative region of the tissue. The observed expression difference was statistically significant (*P* < 0.001) (Figure 2). In the tumor microenvironment, HPRT1 was predominantly expressed in tumor cells.

### Correlation of *HPRT1* with clinical features

**Figure 3.**
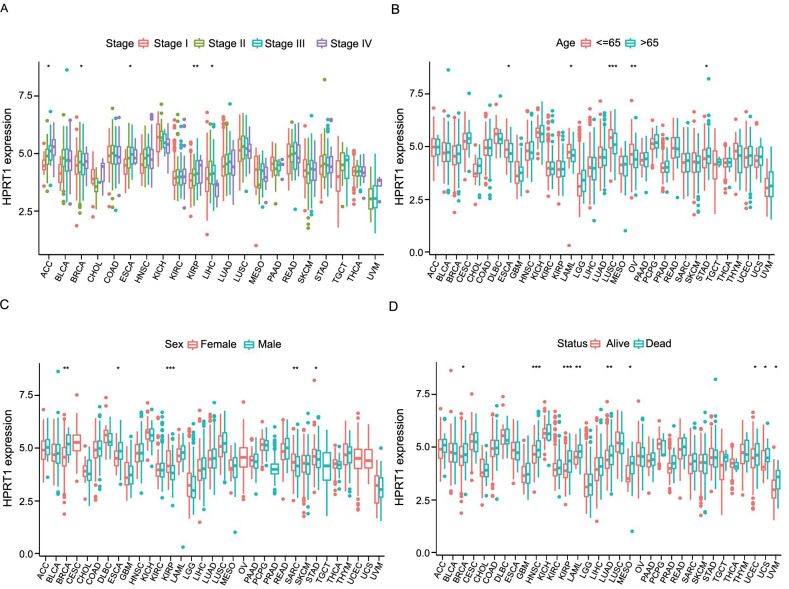
**Relationship between *HPRT1* expression and (A) tumor stage, (B) age distribution, (C) sex and (D) survival status.** *P < 0.05; **P < 0.01; ***P < 0.001. HPRT1: Hypoxanthine phosphoribosyl transferase 1.

The expression level of *HPRT1* varied among patients at different stages. In BRCA, ESCA, and LIHC, the highest expression of *HPRT1* was noted in stage II. In ACC and KIRP, the highest expression was seen in stage IV (Figure 3A). The expression of *HPRT1* was correlated with different age groups. In STAD, older patients exhibited higher *HPRT1* expression. In contrast, younger patients showed higher expression in ESCA, LAML, LUSC, and OV (Figure 3B). Sex differences in *HPRT1* expression were observed: it was higher in women with KIRP, SARC, and STAD, and higher in men with BRCA and ESCA (Figure 3C). Additionally, there was also a correlation between *HPRT1* expression and status. Tumors with high *HPRT1* expression in the deceased group included BRCA, HNSC, KIRP, LAML, LUAD, MESO, UCEC, UCS, and UVM (Figure 3D).

The lack of normal groups for CESC, OV, UCEC, and UCS, as well as the absence of tumor groups for PRAD and TGCT in the sex-based differential expression study of *HPRT1*, precluded analysis of these cancers.

### Correlation between *HPRT1* and the prognosis of patients with cancer

**Figure 4.**
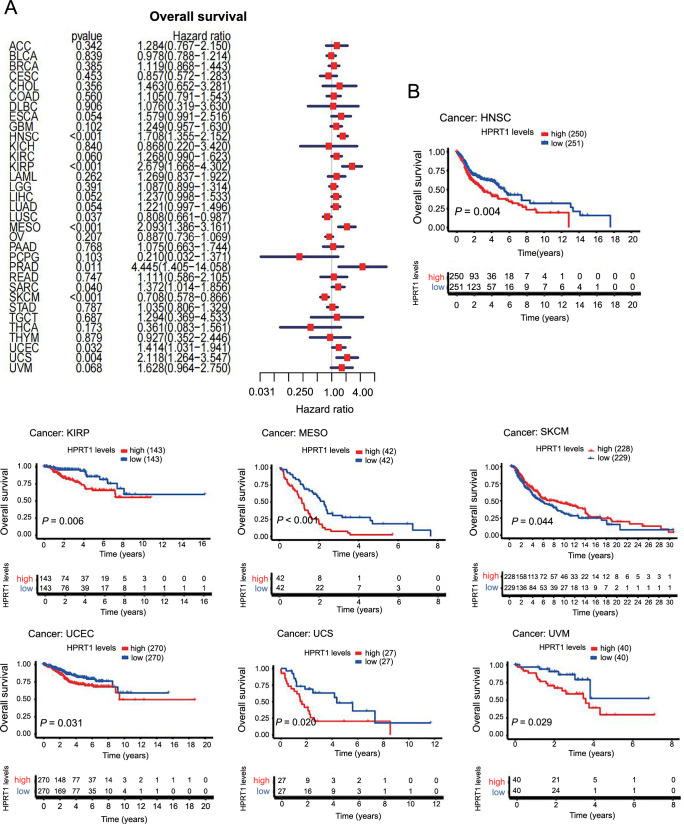
**Association of *HPRT1* expression with OS in cancer patients.** *HPRT1* expression was a risk factor in cancer patients (A), which was associated with shorter OS in patients with multiple cancers (B). (*P* < 0.05 is considered statistically significant). HPRT1: Hypoxanthine phosphoribosyl transferase 1; OS: Overall survival; HNSC: Head-neck squamous cell carcinoma; KIRP: Kidney renal papillary cell carcinoma; MESO: Mesothelioma; SKCM: Cutaneous skin melanoma; UCEC: Uterine corpus endometrial carcinoma; UCS: Uterine carcinosarcoma; UVM: Uveal melanoma.

**Figure 5.**
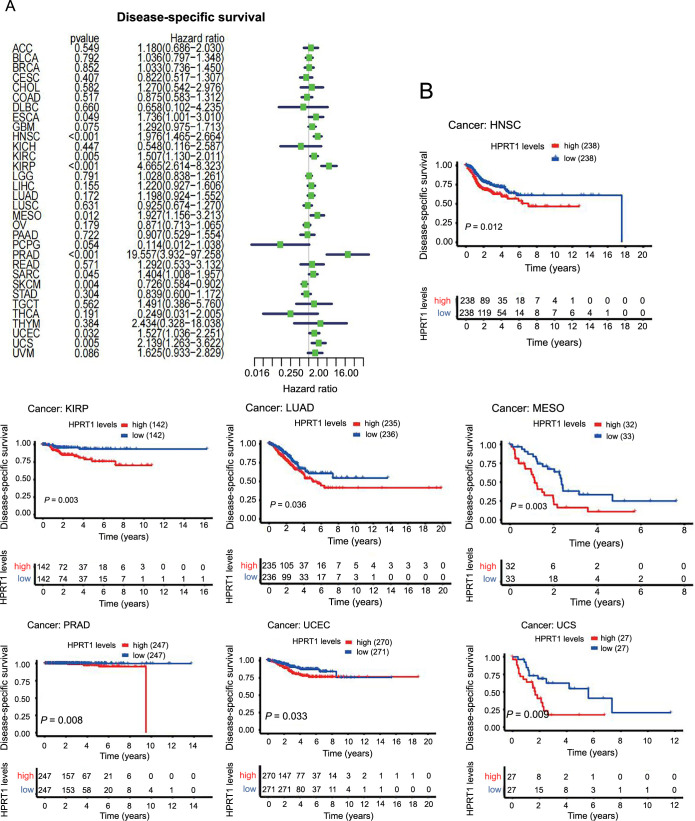
**Association of *HPRT1* expression with disease-specific survival in cancer patients.** *HPRT1* expression was a risk factor in cancer patients (A), and it was associated with shorter disease-specific survival times in patients with multiple cancers (B). (*P* < 0.05 is considered statistically significant). HPRT1: Hypoxanthine phosphoribosyl transferase 1. HNSC: Head-neck squamous cell carcinoma; KIRP: Kidney renal papillary cell carcinoma; LUAD: Lung adenocarcinoma; MESO: Mesothelioma; PRAD: Prostate adenocarcinoma; UCEC: Uterine corpus endometrial carcinoma; UCS: Uterine carcinosarcoma.

**Figure 6.**
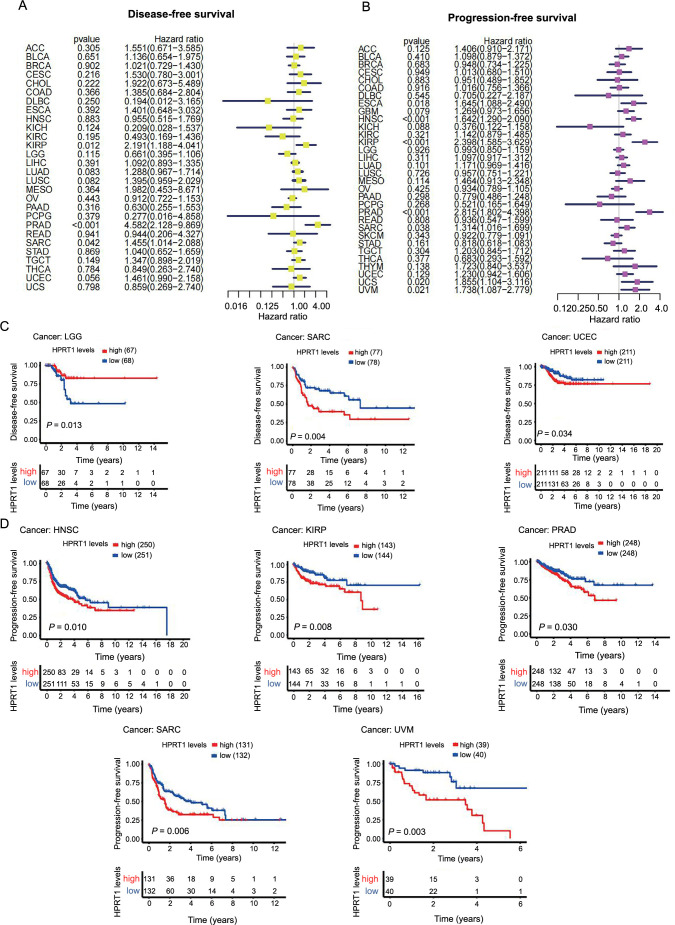
**Association of *HPRT1* expression with DFS and PFS in cancer patients**. *HPRT1* expression was a risk factor in cancer patients (A and B) and was associated with shorther DFS (C) and PFS (D) in patients with multiple cancers. (*P* < 0.05 is considered statistically significant). HPRT1: Hypoxanthine phosphoribosyl transferase 1; DFS: Disease-free survival; PFS: Progression-free survival; LGG: Lower-grade glioma; SARC: Sarcoma; UCEC: Uterine corpus endometrial carcinoma; HNSC: Head-neck squamous cell carcinoma; KIRP: Kidney renal papillary cell carcinoma; PRAD: Prostate adenocarcinoma; UVM: Uveal melanoma.

We first investigated the role of *HPRT1* in pan-cancer prognosis using the Cox model. In HNSC, KIRP, MESO, PRAD, cutaneous skin melanoma (SKCM), UCEC, and UCS, *HPRT1* expression was associated with the OS of patients. *HPRT1* was identified as an unfavorable prognostic factor for OS in HNSC, KIRP, MESO, PRAP, UCEC, and UCS but a favorable prognostic factor in SKCM (Figure 4A). Regarding DSS, *HPRT1* played a protective prognostic role in SKCM but was an unfavorable prognostic factor in HNSC, KIRP, KIRC, MESO, PRAD, SARC, UCEC, and UCS (Figure 5A). For DFS, *HPRT1* was a detrimental prognostic factor for KIRP, PRAD, and SARC (Figure 6A). In terms of PFS, *HPRT1* posed a high-risk gene in ESCA, HNSC, KIRP, PRAD, SARC, UCS, and UVM (Figure 6B).

Subsequently, we analyzed the relationship between *HPRT1* expression and patient outcomes using Kaplan–Meier analysis. The high *HPRT1* expression group had shorter OS in HNSC, KIRP, MESO, SKCM, UCEC, UCS, and UVM (Figure 4B). The high *HPRT1* expression group had shorter DSS in HNSC, KIRP, LUAD, MESO, PRAD, UCEC, and UCS (Figure 5B). The high *HPRT1* expression had shorter DFS in LGG, SARC, and UCEC (Figure 6C). In addition, the high *HPRT1* expression had shorter PFS in HNSC, KIRP, PRAD, SARC, and UVM (Figure 6D).

### The relationship between *HPRT1* and tumor immunity

**Figure 7.**
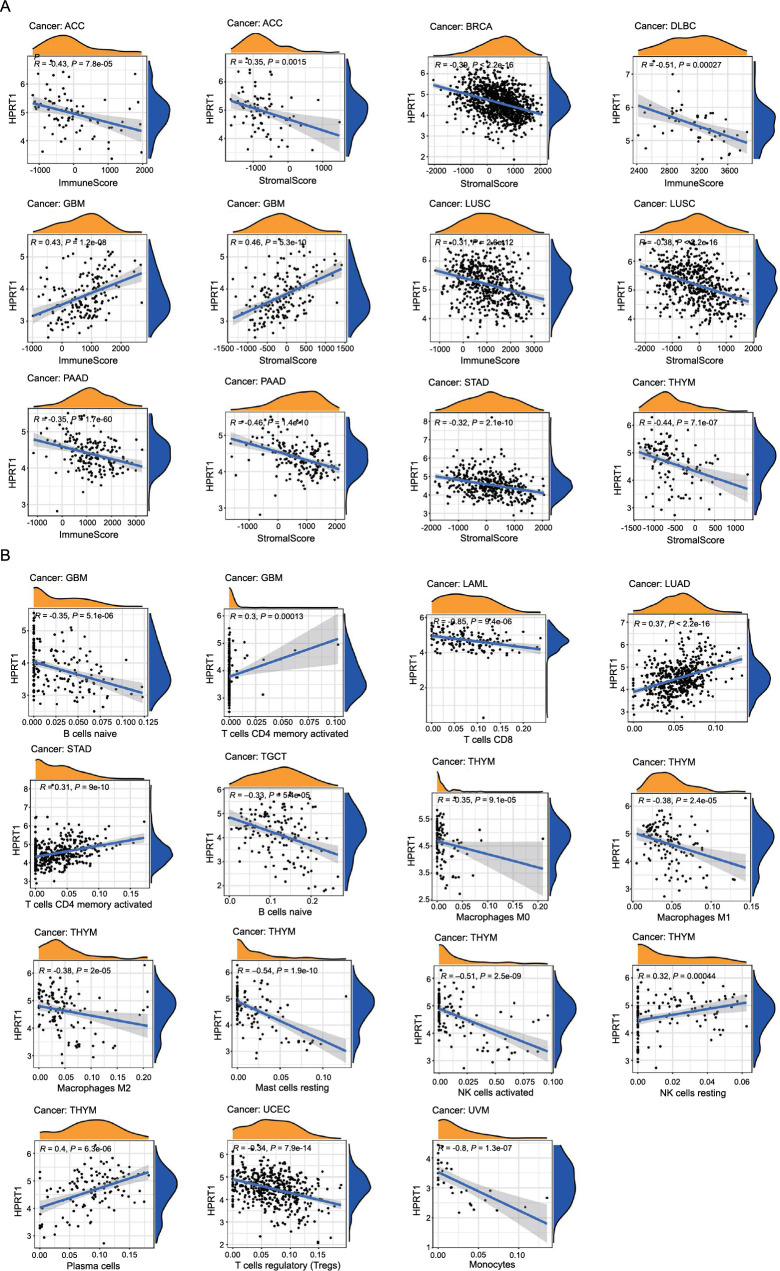
(A) The correlation of *HPRT1* with stromal and immune cell content across eight tumors; (B) The correlation between *HPRT1* and immune cell types in different tumor types. *P* < 0.05 was considered to be correlated. The letter “ρ” is followed by the Spearman correlation coefficient. HPRT1: Hypoxanthine phosphoribosyl transferase 1.

We analyzed the relationship between *HPRT1* and the pan-cancer tumor microenvironment using the ESTIMATE algorithm. *HPRT1* showed a positive correlation with stromal scores in GBM but a negative correlation in ACC, BRCA, LUSC, pancreatic adenocarcinoma (PAAD), STAD, and thymoma (THYM). Furthermore, *HPRT1* was positively correlated with immune scores in GBM but negatively correlated in ACC, DLCA, GBM, LUSC, and PAAD (Figure 7A).

**Figure 8.**
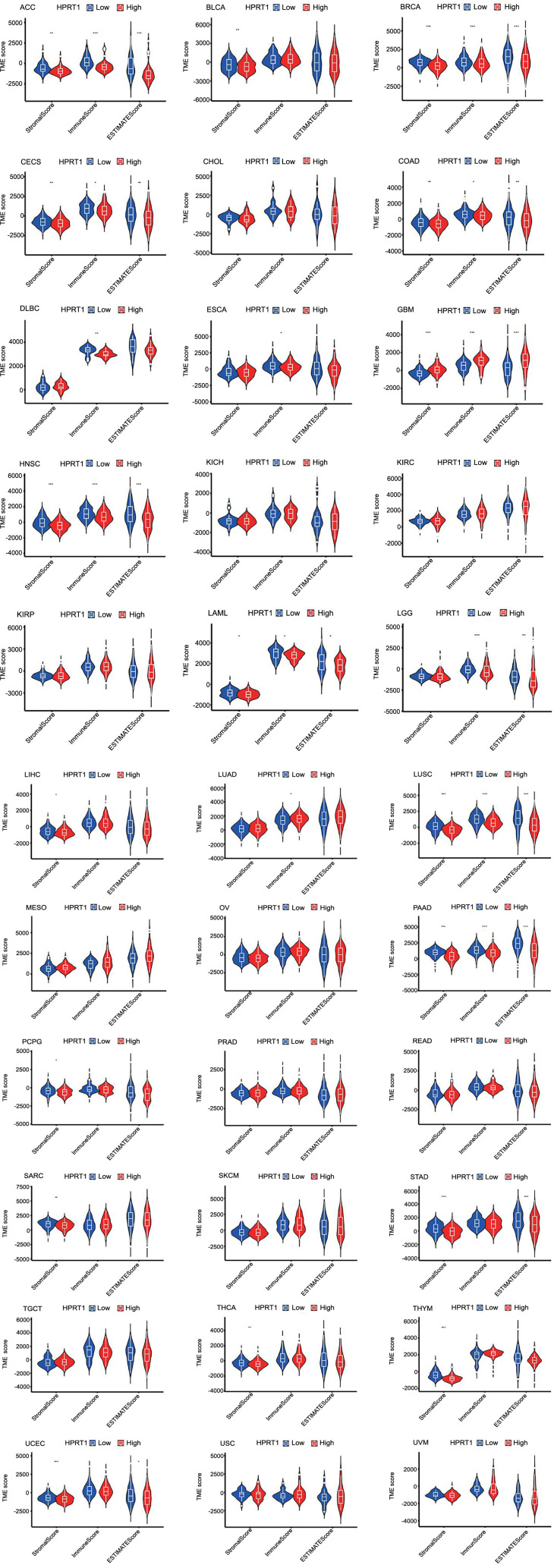
**Relationship between *HPRT1* expression and tumor microenvironment in 33 cancers**. *P < 0.05; **P < 0.01; ***P < 0.001. HPRT1: Hypoxanthine phosphoribosyl transferase 1.

The analysis of the differences in tumor microenvironment between high and low *HPRT1* expression revealed that in cancers, such as ACC, BRCA, CESC, COAD, HNSC, LAML, LUSC, PAAD, STAD, and UCEC, the ESTIMATEScore was lower in the high *HPRT1* expression group compared to the low expression group. This denoted a higher tumor purity, indicating that a higher expression of *HPRT1* corresponded to a greater content of tumor cells in these cases. Contrarily, in GBM, lower levels of tumor cells were found in the high *HPRT1* expression group (Figure 8).

**Figure 9.**
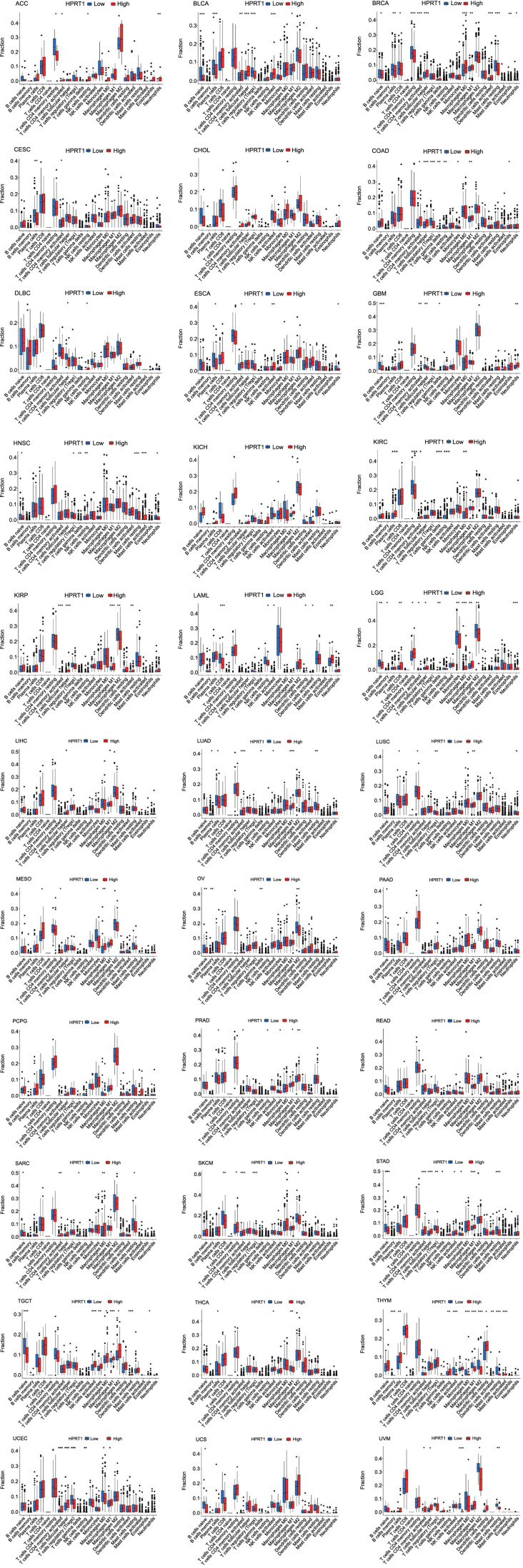
**In 33 cancers, the expression of *HPRT1* was related to 22 immune cell types.** Naive B cells, plasma cells, regulatory T cells, and resting mast cells were more expressed in the low *HPRT1* expression group in various tumors. Activated memory CD4+T cells, T follicular helper cells, M0 macrophages, and M1 macrophages were more expressed in high *HPRT1* expression group of various tumors. *P < 0.05; **P < 0.01; ***P < 0.001. HPRT1: Hypoxanthine phosphoribosyl transferase 1.

The expression of *HPRT1* has been implicated in the immune microenvironment of several cancers, although its role in different cancers may be mediated by affecting different immune cells. Through the analysis of immune cell infiltration, the correlation of *HPRT1* with various immune cells can be observed. In GBM, the expression of *HPRT1* was positively correlated with the content of activated CD4+ T-cells but negatively correlated with naive B-cells. In LUAD, the higher the expression of *HPRT1*, the higher the content of M1 macrophages. In STAD, the expression of *HPRT1* was positively correlated with the content of activated CD4+ T-cells. In LAML, the expression of *HPRT1* was negatively correlated with the content of CD8+ T-cells. In the TGCT, the content of naive B-cells decreased with the expression of *HPRT1*. In UCEC, the expression of *HPRT1* was negatively correlated with the content of regulatory T-cells (Tregs). Similarly, in UVM, the expression of *HPRT1* was negatively correlated with the content of monocytes, and the correlation was high. In THYM, the expression of *HPRT1* has different relationships with different immune cells. The expression of *HPRT1* was positively correlated with the content of resting NK cells and plasma cells, but negatively correlated with resting mast cells, activated NK cells, M0 macrophages, M1 macrophages, and M2 macrophages. *HPRT1* expression had a high correlation with resting mast cells and activated NK cells (Figure 7B). As illustrated in Figure 8, *HPRT1’s* expression, both high and low, was also associated with the content of various immune cells across 33 tumors (Figure 9).

**Figure 10.**
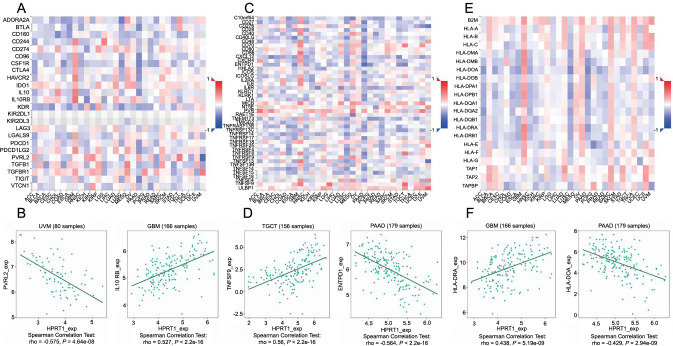
(A) Heatmap of correlations between immunoinhibitor-related genes and *HPRT1* expression; (B) Scatter plot of the correlation between immunoinhibitor-related genes and *HPRT1*; (C) Heatmap of the correlation between immunostimulator-related genes and *HPRT1* expression; (D) Heatmap of the correlation between MHC molecule-related genes and *HPRT1* expression; (E) Scatter plot of the correlation between immunostimulator-related genes and *HPRT1*; (F) Scatter plot of the correlation between MHC molecule-related genes and *HPRT1*. Red: Positive correlation between *HPRT1* expression and immune-related genes at this point. Blue: Inverse correlation between *HPRT1* expression and immune-related genes at this point. HPRT1: Hypoxanthine phosphoribosyl transferase 1; MHC: Major histocompatibility complex.

**Table 2:** Interacting chemicals of *HPRT1* based on CTD

**Chemical name**	**ID**	**Interaction action**	**Chemical name**	**ID**	**Interaction action**
1-Nitrosopyrene	C044707	Increases mutagenesis	Estradiol	D004958	Increases expression
2-Amino-1-methyl-6-phenylimidazo(4,5-b)pyridine	C049584	Increases mutagenesis	Ethylnitrosourea	D005038	Increases mutagenesis
Acrylamide	D020106	Increases mutagenesis	Glycidamide	C071834	Increases mutagenesis
3-Chloro-4-(dichloromethyl)-5-hydroxy-2(5H)-furanone	C054121	Increases mutagenesis	Perfluorooctanoic acid	C023036	Increases expression
Bisphenol A	C006780	Decreases expression	Potassium dichromate	D011192	Increases mutagenesis
Chromium hexavalent ion	C074702	Increases mutagenesis	Tetrachlorodibenzodioxin	D013749	Increases expression
Ethinyl estradiol	D004997	Increases expression	Tetrachlorodibenzodioxin	D013749	Affects expression
Ethyl methanesulfonate	D005020	Increases mutagenesis	Thioguanine	D013866	Decreases response to substance
Tretinoin	D014212	Decreases expression	1,8-Dinitropyrene	C033310	Increases mutagenesis
Valproic acid	D014635	Increases expression	4-Nitroquinoline-1-oxide	D015112	Increases mutagenesis
1,3-Butadiene	C031763	Increases mutagenesis	Benzo(a)pyrene	D001564	Increases mutagenesis
1,3-Butadiene	C031763	Increases mutagenesis	Hydrogen peroxide	D006861	Increases mutagenesis
Benzo(a)pyrene	D001564	Increases expression	Nitric oxide	D009569	Increases mutagenesis
Bisphenol A	C006780	Affects expression	Pirinixic acid	C006253	Increases expression
Cyclophosphamide	D003520	Increases mutagenesis	Styrene	D020058	Increases mutagenesis

CTD: Comparative toxicogenomics database; HPRT1*:* Hypoxanthine phosphoribosyl transferase 1.

We further analyzed the association between *HPRT1* and immunoinhibitors, immunostimulators, and MHC molecules in the TISIDB database. Correlation heatmaps revealed that in most pan-cancer tumors, *HPRT1* expression positively correlated with immunoinhibitors, immunostimulators, and MHC molecules. Then, we identified the darkest red and deepest blue dots in the three heatmaps and created separate scatter plots. For instance, in GBM, *HPRT1* positively correlated with interleukin 10 receptor subunit beta (*IL10RB*). In UVM, there was a negative correlation between *HPRT1* and the poliovirus receptor-related 2 (*PVRL2*) gene (Figure 10A and 10B). In a correlation analysis between immunostimulators and *HPRT1*, we observed a positive correlation between the expression of *HPRT1* and tumor necrosis factor superfamily member 9 (*TNFSF9*) in TGCTs. In PAAD, *HPRT1* and ectonucleoside triphosphate diphosphohydrolase-1 (*ENTPD1*) were negatively correlated (Figure 10C and 10D). In the correlation analysis with MHC molecules, *HPRT1* in GBM positively correlated with *HLA-DRA*, whereas in PAAD, *HPRT1* inversely correlated with *HLA-DOA*. Notably, in all three heatmaps, *HPRT1* in GBM had a significant and positive correlation with most immune genes, while in LUSC and PAAD, *HPRT1* was significantly negatively correlated with most immune genes (Figure 10E and 10F).

**Figure 11.**
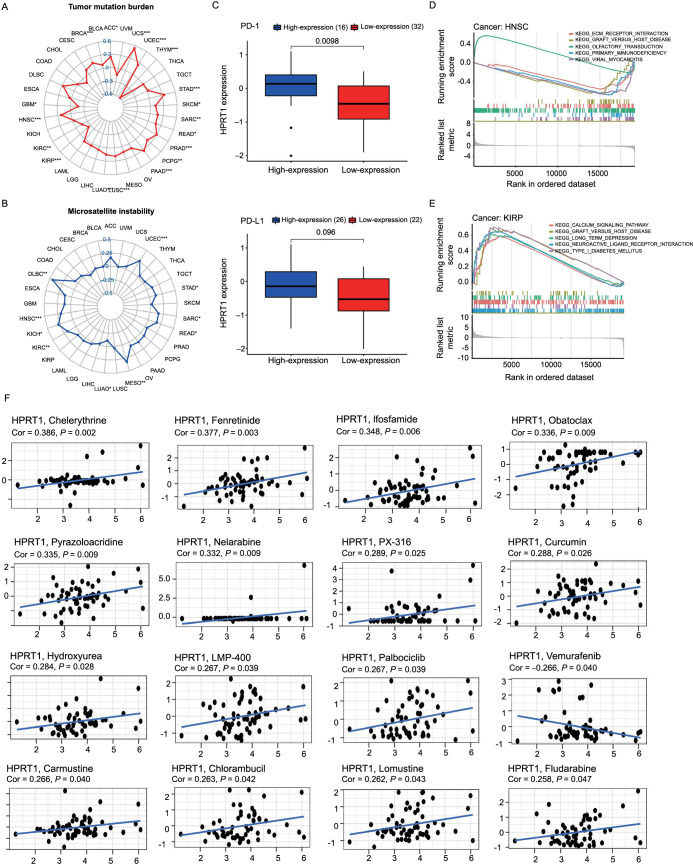
(A) Radar plot showing the association of *HPRT1* and TMB; (B) Radar plot showing the association of *HPRT1* and MSI; (C) The GEO dataset validated the correlation analysis of *HPRT1* and PD-1/PD-L1 (*P* < 0.05 is considered statistically significant); (D and E) GSEA results were based on KEGG datasets in HNSC and KIRP; (F) Sensitive drugs associated with *HPRT1* expression (*P* < 0.05 is considered statistically significant). *P < 0.05; **P < 0.01; ***P < 0.001. HPRT1: Hypoxanthine phosphoribosyl transferase 1; TMB: Tumor mutation burden; MSI: Microsatellite instability; GEO: Gene expression omnibus; GSEA: Gene set enrichment analysis; KEGG: Kyoto encyclopedia of genes and genomes; HNSC: Head-neck squamous cell carcinoma; KIRP: Kidney renal papillary cell carcinoma; PD-1: Programmed cell death-1; PD-L1: Programmed cell death protein-1.

TMB correlation analysis showed that *HPRT1* expression was correlated with TMB of most cancers. In ACC, BRCA, HNSC, LUAD, LUSC, PAAD, PCPG, PRAD, READ, SARC, SKCM, STAD, UCEC, and UCS, *HPRT1* expression was positively correlated with patient TMB. Conversely, in GBM, KIRC, KIRP, and THYM, *HPRT1* expression was negatively correlated with TMB (Figure 11A). There was also a correlation observed between *HPRT1* expression and MSI. *HPRT1* expression was positively correlated with MSI in DLBC, HNSC, KICH, KIRC, MESO, READ, SARC, STAD, and UCEC. In LUAD, *HPRT1* expression was negatively correlated with MSI (Figure 11B).

Using the GSE107764 dataset (*n* ═ 48), we verified the relationship between *HPRT1* and *PD-1/PD-L1*. *HPRT1* was found to be positively correlated with *PD-1*, but not with the *PD-L1* expression. There was a significant difference in *HPRT1* expression between the high and low expression groups of *PD-1* (*P* < 0.05) (Figure 11C). This proves that *HPRT1* is related to immunotherapy.

### GSEA enrichment analysis

We analyzed *HPRT1*-related signaling pathways in 33 tumors by GSEA to identify differentially activated pathways in multiple malignancies when *HPRT1* is highly expressed. Considering the strong correlation of *HPRT1* with HNSC and KIRP, we focused on the potential pathways and roles of *HPRT1* signaling in these two tumors (Figure 11D and 11E).

**Table 3:** Relationship of *HPRT1* with genes via chemical interaction, based on CTD

**Gene**	**Similarity index**	**Common interacting chemicals**
*DNAJC3*	0.223	64
*PDIA3*	0.22082	70
*ISYNA1*	0.21691	59
*PGRMC1*	0.20818	56
*IGFBP2*	0.20732	68
*OAT*	0.20468	70
*NDRG2*	0.20423	58
*CARHSP1*	0.20301	54
*PDCD4*	0.20186	65
*DUT*	0.20141	57
*CCT3*	0.2	53
*TMPO*	0.2	59
*ALCAM*	0.19932	59
*SFXN1*	0.1992	50

CTD: Comparative toxicogenomics database; HPRT1*:* Hypoxanthine phosphoribosyl transferase 1.

### Drug sensitivity analysis

This study involved screening for drugs that are sensitive to cells with high *HPRT1* expression, using 263 drugs approved by the U.S. FDA. Finally, the top 16 most relevant drugs were screened, including chelerythrine, fenretinide, ifosfamide, and obatoclax (Figure 11F).

### Chemical and gene interactions of *HPRT1*

Data from the CTD database demonstrated that *HPRT1* was associated with 50 chemicals. Seven of these chemicals were found to upregulate *HPRT1*, while two could downregulate it. Eighteen chemicals have been shown to increase mutagenesis in *HPRT1*, and one chemical reduced reaction of *HPRT1* to substances. In addition, two chemicals affected the expression of *HPRT1*, but their specific roles were unclear (Table 2). Furthermore, the first 20 relationships between *HPRT1* and other genes were identified through chemical associations. The results showed that *HPRT1* was highly correlated with DnaJ homolog subfamily C member 3 (*DNAJC3*), protein disulfide-isomerase A3 (*PDIA3*), inositol-3-phosphate synthase 1 (*ISYNA1*), progesterone receptor membrane component 1 (*PGRMC1*), insulin-like growth factor-binding protein 2 (*IGFBP2*), and ornithine aminotransferase (*OAT*) (Table 3).

**Figure 12.**
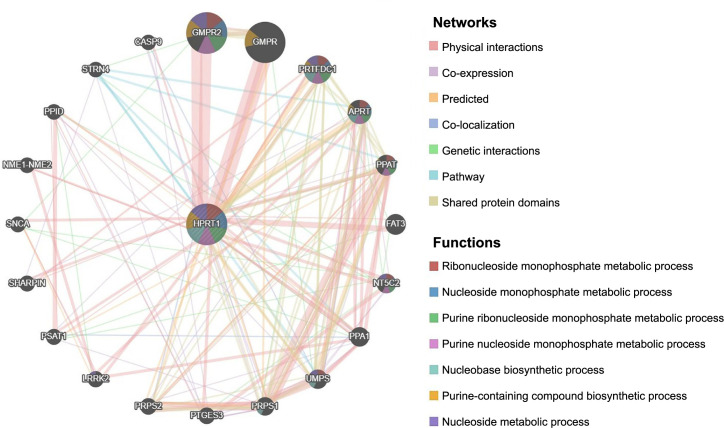
**Gene–gene interaction networks for *HPRT1* and similar genes.** The genes highly associated with multiple functions of *HPRT1* include *GMPR2*, *PRTFDC1*, *APRT*, *PPAT*, *NT5C2*, and *UMPS*. HPRT1: Hypoxanthine phosphoribosyl transferase 1; GMPR2: Guanosine monophosphate reductase 2; PRTFDC1: Phosphoribosyl transferase domain containing 1; APRT: Adenin-Phosphoribosyltransferase; PPAT: Phosphoribosyl pyrophosphate amidotransferase; NT5C2: Cytosolic 5′ nucleotidase II; UMPS: Uridine 5’-monophosphate synthase.

GeneMANIA was employed to construct gene–gene interaction networks for *HPRT1* and similar genes. The results showed that the 20 genes were closely associated to *HPRT1*, with guanosine monophosphate reductase 2 (*GMPR2*) being the most strongly connected. Functionally, *HPRT1* and its related genes were significantly linked to ribonucleoside monophosphate, nucleoside, and purine nucleoside monophosphate metabolic processes (Figure 12).

### HPRT1 expression in HNSC

#### Immunotherapy analysis of HPRT1

**Figure 13.**
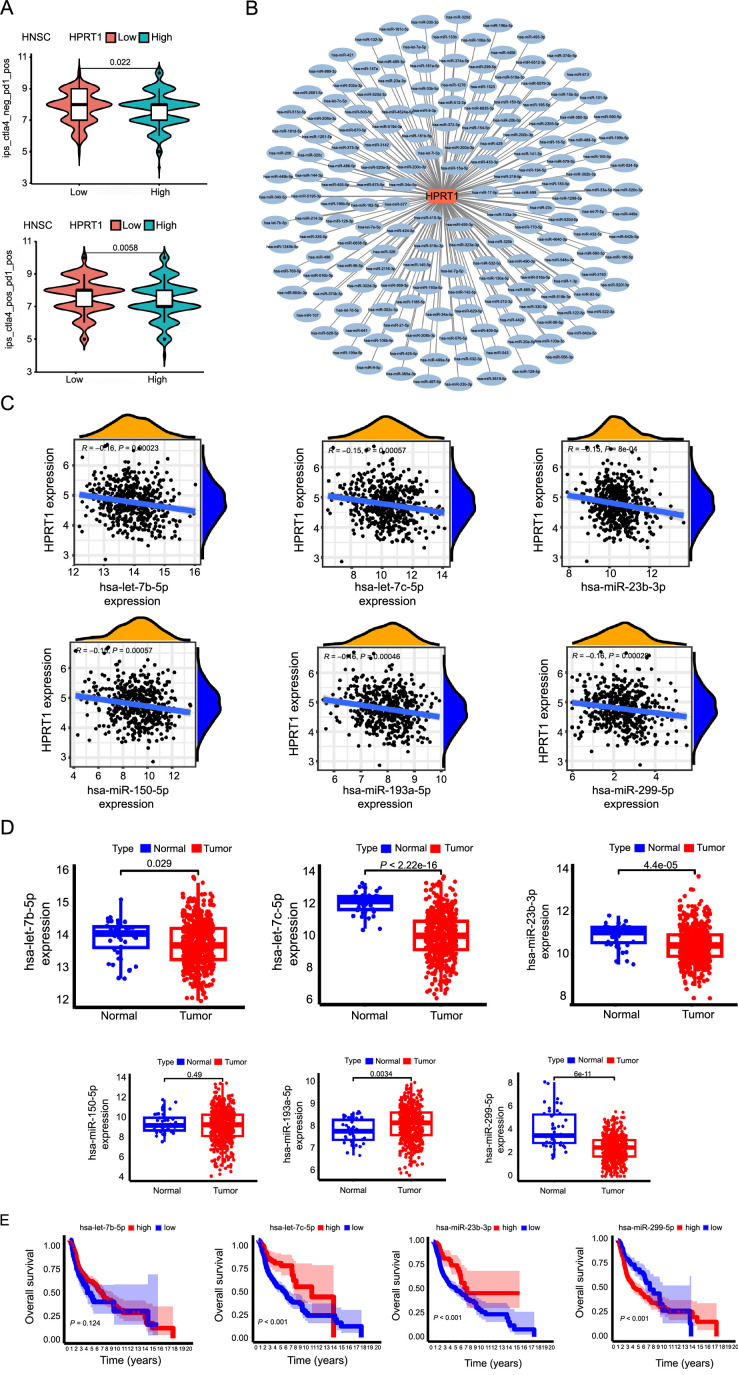
(A) Correlation between *HPRT1* expression and immunotherapy in HNSC data; (B) *HPRT1*-associated miRNA–mRNA regulatory network diagram; (C) *HPRT1* and miRNA correlation analysis; (D) Analysis of the difference between the expression of miRNAs in tumor and normal tissues; (E) Relationship between miRNA and survival prognosis (*P* < 0.05 is considered statistically significant). HPRT1: Hypoxanthine phosphoribosyl transferase 1; HNSC: Head and neck squamous cell carcinoma.

We performed an immunotherapeutic analysis of HNSC. The ordinate represents the immune score, hence a higher score indicates a better patient response to receiving immunotherapy. The results demonstrated differences in immunotherapy response between the high and low expression groups of HNSC, with the low expression group showing higher outcome scores. Notably, this indicates that the low expression group exhibited a better effect when receiving immunotherapy. Interestingly, patients had better outcomes when treated with a combination of PD-1 and CLA-4 (Figure 13A).

#### HPRT1-related ceRNA networks in HNSC

Cytoscape software was used to build *HPRT1*-related miRNA–mRNA regulatory network diagrams (Figure 13B). Through correlation analysis, identified miRNAs negatively correlated with *HPRT1*: hsa-let-7c-5p, hsa-miR-23b-3p, hsa-miR-299-5p, hsa-let-7b-5p, hsa-miR-150-5p, and hsa-miR-193a-5p (Figure 13C). The difference between the expression of miRNA in tumor and normal tissues showed that the following hsa-let-7c-5p, hsa-miR-23b-3p, hsa-miR-299-5p, and hsa-let-7b-5p were downregulated in tumor tissues and had significant expression differences between normal and tumor tissues, which met our screening conditions. Due to the high expression of hsa-miR-193a-5p in the tumor group and the no significant difference in expression of hsa-miR-150-5p between tumor group and the normal group, these two miRNAs were excluded (Figure 13D). Consequently, hsa-miR-23b-3p, hsa-miR-299-5p, and hsa-let-7c-5p were selected based on their association with survival prognosis and differential expression. Hsa-let-7b-5p was not associated with survival prognosis, so it was excluded (Figure 13E). With the aforementioned steps, we screened hsa-miR-23b-3p, hsa-miR-299-5p, and hsa-let-7c-5p.

**Figure 14.**
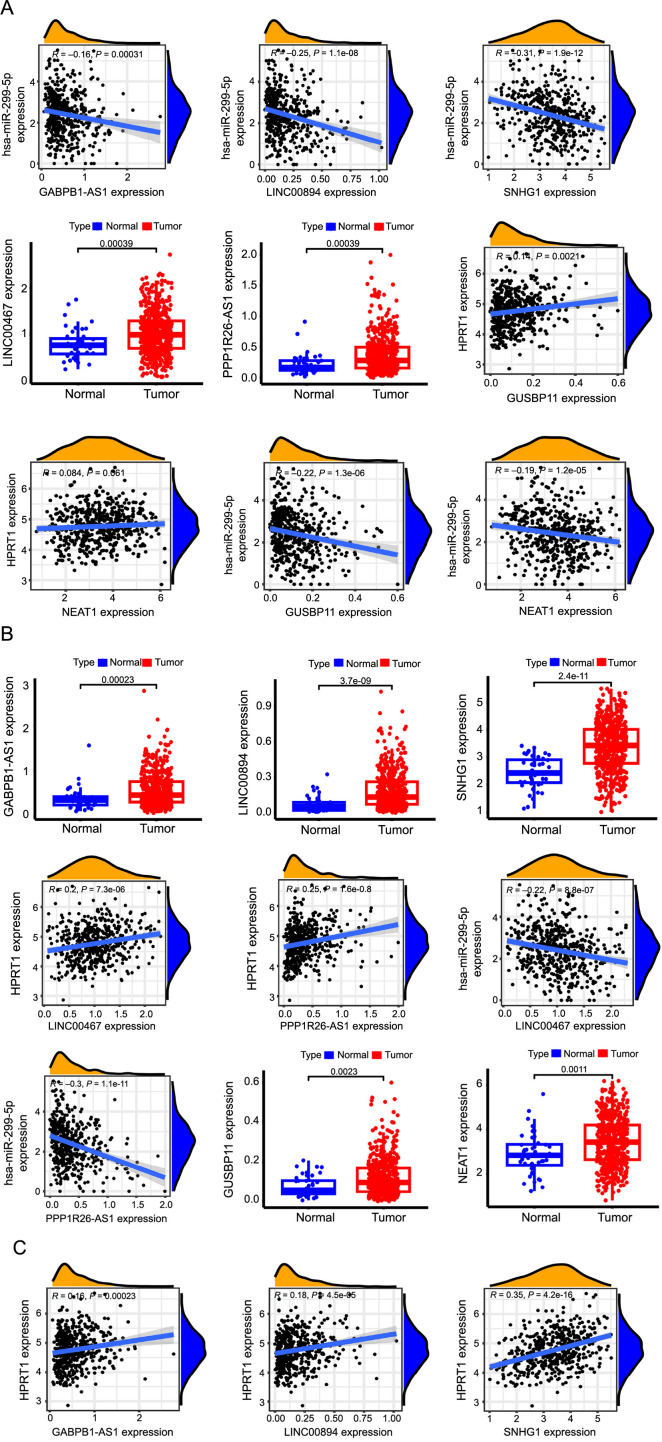
(A) Correlation analysis of hsa-miR-299-5p and lncRNA; (B) Analysis of differential expression of lncRNA in tumor and normal tissues; (C) Correlation analysis of *HPRT1* and screening lncRNAs (*P* < 0.05 is considered statistically significant). HPRT1: Hypoxanthine phosphoribosyl transferase 1; lncRNA: Long non-coding RNA.

**Figure 15.**
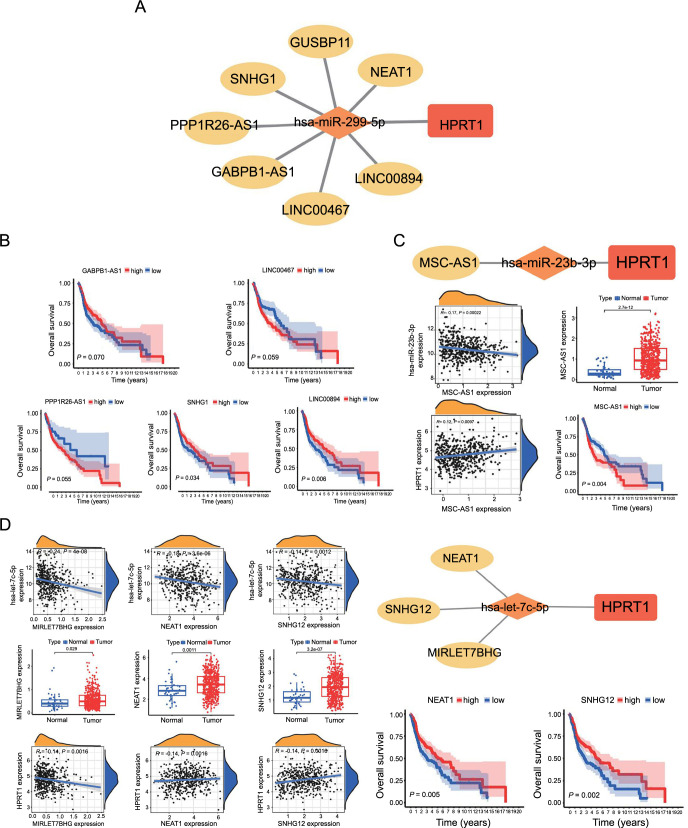
(A) ceRNA network based on hsa-miR-299-5p; (B) Survival analysis of lncRNAs associated with hsa-miR-299-5p in tumor tissues; (C) Integrated analysis of ceRNA network of hsa-miR-23b-3p, along with correlation analysis between hsa-miR-23b-3p and lncRNA and between lncRNA and HPRT1, including expression difference analysis of lncRNA, and the survival curve analysis of lncRNA; (D) The ceRNA network of hsa-let-7c-5p, the correlation analysis between hsa-let-7c-5p and lncRNA and between lncRNA and *HPRT1*, the expression difference analysis of lncRNA and the survival curve analysis of lncRNA (*P* < 0.05 is considered statistically significant). HPRT1: Hypoxanthine phosphoribosyl transferase 1; ceRNA: competitive endogenous RNA; lncRNA: long non-coding RNA.

LncRNAs associated with hsa-miR-299-5p were sourced from the starBase database. These lncRNAs were analyzed and screened for their significant negative correlation with hsa-miR-299-5p as follows: NEAT1, LINC00894, PPP1R26-AS1, SNHG1, GABPB1-AS1, GUSBP11, and LINC00467, which are divergent lncRNAs (Figure 14A). Cytoscape software was used to construct a ceRNA network diagram of *HPRT1* (Figure 15A). The differential expression analysis of lncRNAs in tumor and normal tissues showed that the above seven lncRNAs were significantly different. All of them were highly expressed in tumor tissues (Figure 14B). Correlation analysis of *HPRT1* and the above seven lncRNAs, demonstrated that six of them exhibited a significant positive correlation with *HPRT1*, including LINC00894, PPP1R26-AS1, SNHG1, GABPB1-AS1, GUSBP11, and LINC00467. The relationship between NEAT1 and *HPRT1* was insignificant and, therefore, excluded (Figure 14C). The lncRNAs associated with survival prognosis were LINC00894 and SNHG1. In contrast, LINC00467, PPP1R26-AS1, and GABPB1-AS1 were excluded (Figure 15B). Similarly, the same analysis steps were performed for hsa-miR-23b and hsa-let-7c-5p to find lncRNAs that met the conditions (Figure 15C and 15D).

Through the aforementioned process, the final selection of five axes was determined, including LINC00894-hsa-miR-299-5p-HPRT1, SNHG1-hsa-miR-299-5p-HPRT1, SNHG12-hsa-let-7c-5p-HPRT1, NEAT1-hsa-let-7c-5p-HPRT1, and MSC-AS1-hsa-miR-23b-5p-HPRT1.

#### Expression of HPRT1 and PD-1 in oral squamous cell carcinoma cell line

**Figure 16.**
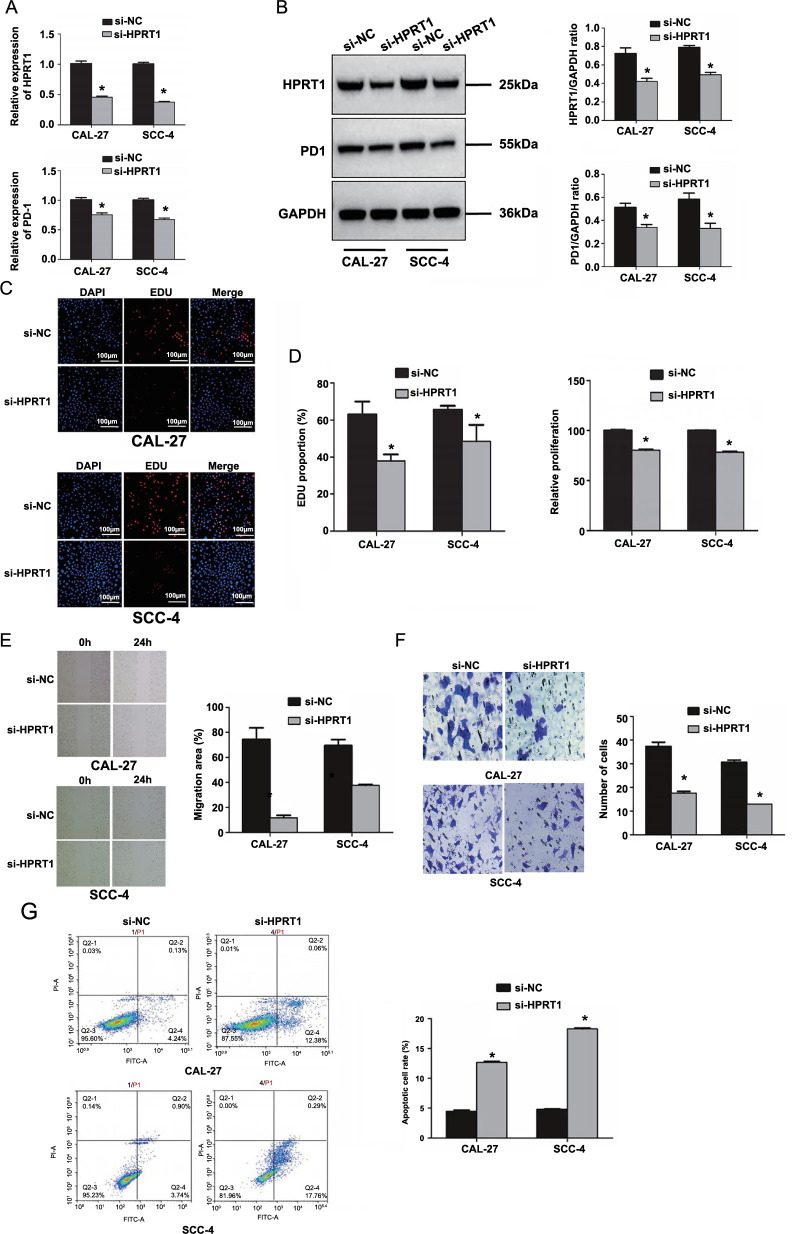
(A) Differential analysis of the expression levels of *HPRT1* and PD-1 in the si-NC and si-HPRT1 groups in RT-qPCR; (B) Differential analysis of the expression levels of HPRT1 and PD-1 in the si-NC and si-HPRT1 groups in Western blot; (C) The EdU experiment confirmed the difference in proliferation ability between the si-NC and si-HPRT1 groups; (D) The CCK-8 experiment confirmed the difference in proliferation ability between the si-NC and si-HPRT1 groups; (E) The migration ability of cells in the si-NC and si-HPRT1 groups was compared in the cell scratch experiment; (F) The invasiveness of cells in the si-NC and si-HPRT1 groups was compared in the transwell experiment; (G) The apoptosis of cells in the si-NC and si-HPRT1 groups was compared by flow cytometry (*P < 0.05 is considered statistically significant). HPRT1: Hypoxanthine phosphoribosyl transferase 1; si-NC: Small interfering non-coding RNA; RT-qPCR: Reverse transcriptase polymerase chain reaction; PD-1: Programmed cell death-1; CCK-8: Cell Counting Kit-8; EdU: 5-Ethyl-2’-deoxyridine.

The knockdown effect of *HPRT1* was verified using RT-qPCR and western blotting methods. *HPRT1* was successfully knockdown in the si-HPRT1 group in CAL-27 and SCC-4 cell lines (*P* < 0.05). The expression level of PD-1 in the si-NC group was significantly higher than that in si-HPRT1 (*P* < 0.05), indicating a positive correlation between PD-1 and *HPRT1* expression (Figure 16A and 16B).

#### Effect of HPRT1 on proliferation, migration, and apoptosis of oral squamous cell carcinoma cells

CCK-8 and EdU were used to detect the proliferation of oral squamous cell carcinoma cells in each group. As a result, compared with the CAL-27 and SCC-4 cells in the si-NC group, the cell proliferation in the si-HPRT1 group decreased sharply (both *P* < 0.05) (Figure 16C and 16D).

Cell migration, assessed via scratch assay, showed reduced migration ability in the si-HPRT1 group as compared to the CAL-27 and SCC-4 in the si-NC group (both *P* < 0.05) (Figure 16E).

The transwell experiment was used to detect the invasiveness of cells. The results showed that, compared with the CAL-27 and SCC-4 cells in the si-NC group, the invasive ability of the si-HPRT1 group decreased (both *P* < 0.05) (Figure 16F).

Flow cytometry was used to detect cell apoptosis, and it revealed a significant increase in apoptotic cells in the si-HPRT1 group compared to the si-NC group (*P* < 0.05) (Figure 16G).

## Discussion

HPRT1 is an enzyme in the DNA salvage pathway responsible for circulating guanosine triphosphate, which is involved in regulating the synthesis of purines in the cell cycle. This enzyme is implicated in modulating immune mechanisms within the tumor microenvironment. Elevated *HPRT1* levels in tumors may contribute to immune evasion, fostering, and immunosuppressive tumor microenvironment [24]. *HPRT1* expression is associated with the occurrence and progression of multiple cancer types, such as BRCA, HNSC, LUAD, LUSC, PRAD, and UCEC [7–11]. However, a comprehensive pan-cancer analysis of *HPRT1* has not been fully investigated. Thus, our study focused on the functional role of *HPRT1* in a variety of tumors, with a particular emphasis on HNSC. We explored tumors RNA expression levels in relation to clinical features and their impact on prognosis. We also conducted GO and Kyoto Encyclopedia of Genes and Genomes (KEGG) analysis of HNSC, along with examining tumor immunity. Additionally, we delved into the upstream lncRNA–miRNA network that regulates *HPRT1* expression in HNSC and validated the relationship between HPRT1 and PD-1.

First, there were significant differences in *HPRT1* expression and activity across multiple cancers compared to paired normal tissues. Unfortunately, the lack of normal group samples in the TCGA database limited our analysis in cancers, such as ACC, DLBC, LAML, LGG, MESO, OV, TGCT, UCS, and UVM. This gap warrants further investigation as the dataset expands. Our study revealed that in most cancer types, *HPRT1* expression did not significantly vary by sex, age, tumor stage, or status. However, age was significantly associated with *HPRT1* expression in LUSCs. Sex and status were significantly associated with the expression of *HPRT1* in KIRP. By enzyme-linked immunosorbent assay, Feng et al. [25] found that HPRT1 was associated with worse DFS and OS in lung cancer patients. In addition, in UCEC, the survival rate of patients with high expression of *HPRT1* was significantly reduced compared to that of patients with low expression [11]. Using a univariate Cox regression analysis method, Ye et al. [26] revealed a significant association of *HPRT1* with OS in oral squamous cell carcinoma. In Li et al.’s study [24], analysis showed that the upregulation of genes, such as *CD276*, *LDHB*, *SLC3A2*, *EGFR*, *SLC7A5*, and *HPRT1*, is potentially detrimental to the progression of HNSC. Combined with further survival analysis, we confirmed that the expression of *HPRT1* in HNSC is significantly associated with OS, PFS, and DSS but not with DFS. The expression of HPRT1 in KIRP is significantly related to OS, DSS, and DFS but not to PFS. *HPRT1* has significant prognostic significance in some cancers, suggesting its potential value in cancer treatment and prediction. Ahmadi et al. [11] found that the HPRT1 gene may mediate the development of HNSC through the cell cycle and apoptosis-related pathways. Wu et al. [1] found that HPRT1 regulates cisplatin resistance to cisplatin in oral squamous cell carcinoma by positively regulating the expression of matrix metallopeptidase and activating the phosphoinositide 3-kinase/AKT pathway. We used GSEA to show that *HPRT1* expression is significantly associated with a range of signaling and immunomodulation-related pathways, revealing possible molecular mechanisms of *HPRT1*. In HNSC, *HPRT1* is involved in cell-specific interactions with the extracellular matrix (ECM), associated with biological behaviors, such as migration, differentiation, proliferation, and apoptosis of HNSC. In KIRP, HPRT1 is involved in the calcium signaling pathway, which is closely related to cell signaling.

Tumor immunity refers to the sum of the complex interactions between various cells in the tumor microenvironment [27]. The tumor microenvironment consists of malignant cells, parenchymal cells, fibroblasts, mesenchymal cells, and immune cells [28]. All immune cell subsets can be found in tumors, but their respective density, function, and tissue vary from tumor to tumor [29]. The infiltration of immune cells in the tumor microenvironment is an essential factor affecting the effectiveness of tumor immunotherapy. *HPRT1* expression is associated with the immune microenvironment of certain cancers, and its role in various cancers may be by affecting various immune cells. The expression of *HPRT1* was associated with various immune cell subsets; infiltration levels of B-cells, CD4+ T-cells, CD8+ T-cells, macrophages, neutrophils, and dendritic cells were inversely correlated with *HPRT1* expression. The elevation of *HPRT1* in tumors can significantly affect the activation and penetration of immune cells, promoting the production of adenosine. By controlling adenosine production, *HPRT1* upregulation is able to suppress the immune microenvironment of malignant tumors [30]. Li et al. [24] found that in HNSC, the expression level of *HPRT1* was inversely correlated with B-cells, CD4+ T-cells, and CD8+ T-cells. Zhuang and Gao [10] used the CiberSort algorithm to find that in LUSC, the expression of *HPRT1* was inversely proportional to the number of B-cells, CD4+ T-cells, CD8+ T-cells, macrophages, neutrophils, and dendritic cells.

Cellular immunity mediated by T lymphocytes plays a major role in the antitumor immune mechanism. CD8+ T-cells mainly exert an antitumor effect, and the role of CD4+ T-cells is to promote their activation and proliferation. These two cell types can synergistically fight tumors [27]. In triple-negative breast cancer, higher CD4/CD8 ratios and CD30 expression levels correlate with poor prognosis and lower OS rate. However, increased CD8+ expression suggests a better prognosis and improved OS [31]. The abnormal number and function of T lymphocyte subsets in patients with malignant tumors lead to immune dysfunction, which, in turn, promotes tumor development [27]. B-cells form antigen-specific immune responses in the tumor microenvironment by presenting antigens to CD4+ and CD8+ T-cells. Tumor-infiltrating B-cells may play a role in promoting and inhibiting tumor formation, depending on the composition of the tumor microenvironment, B-cells the phenotype and produced antibodies [32]. We observed that *HPRT1* was negatively correlated with the content of naive B-cells in GBM but positively correlated with activated memory CD4+ T-cells. In LAML, *HPRT1* is negatively correlated with CD8+ T-cells. In TGCTs, *HPRT1* is negatively correlated with naive B-cells. Han et al. [33] found that the number of peripheral CD4+ T and CD8+ T lymphocytes in STAD patients can reflect the infiltration status of these lymphocytes in cancer and normal adjacent tissues to a certain extent, and can preliminarily predict immunotherapy response. Through our study analysis, *HPRT1* was positively correlated with activated memory CD4+ T-cells in STAD, and there was a significant difference in expression between the *HPRT1* high-expression and low-expression groups of tumors.

Macrophages play an important part in the tumor microenvironment, influencing cancer cell proliferation, metastasis, and immunosuppression. Additionally, they can mediate the phagocytosis and cytotoxic tumor killing of cancer cells [34]. In malignant tissues, plasma cells are small in number but can produce large amounts of cytokines and antibodies. These antibodies promote antitumor immunity by enhancing antigen presentation by dendritic cells [32]. NK cells infiltrate the lymph nodes of primary and metastatic tumors, detect early signs of tumor transformation or infection, and respond quickly – killing tumor cells [35]. Resting mast cells are particularly important for the treatment of cancer, participating in tissue homeostasis through continuous sampling of the microenvironment [36]. In LUAD, *HPRT1* is positively correlated with M1 macrophages. In THYM, *HPRT1* is associated with various immune cells, of which the expression of *HPRT1* is positively correlated with resting NK cells and plasma cells and negatively correlated with M0 macrophages, M1 macrophages, M2 macrophages, resting mast cells, and activated NK cells. Monitoring the changes in tumor immune cells is of great significance to reflect the immune function and generate prognostic judgments in a timely manner and to observe the curative effect of tumor patients [37].

TMB is defined as the total number of somatic coding mutations associated with the emergence of neoantigens that trigger antitumor immunity and is used to select patients who benefit from immune checkpoint inhibitor therapy [38]. MSI is caused by a DNA mismatch repair (MMR) system defect. Deletion of the MMR gene in tumor cells or defects in the process of replication repair increases the likelihood of gene mutation. MSI is associated with tumor progression and can be a positive predictor of immunotherapy efficacy [39]. In the current study, these two immune checkpoint-blocking therapeutic biomarkers were significantly associated with *HPRT1* in many cancers. There was a significant positive correlation between BRCA, LUSC, PAAD, PRAD, and UCS and *HPRT1* in TMB, while KIRP and THYM were significantly positively correlated with TMB. MSI and TMB were positively correlated with HNSC, READ, SARC, STAD, and UCEC. *HPRT1* may be closely related to ICBT in these tumors. An interesting result was found: in the three heatmaps of immunosuppressants, immunostimulants, and MHC molecules, most of the immune genes in GBM were positively correlated with *HPRT1*. Most immune genes in PAAD and LUSC were significantly and negatively correlated with *HPRTl*. In past immunotherapy explorations, the role of *HPRT1* in PAAD appears to have been overlooked, requiring further immune-related research.

Previous studies have investigated the link between *HPRT1* gene expression profiles and drug sensitivity. Through the GSCALite database, it was displayed that overexpression of *HPRT1* indicated resistance to abiraterone and sensitivity to several drugs like tozasertib and teniposide. This study demonstrated that the elevated expression of the *HPRT1* gene is correlated with the progression of HNSC; thus, this gene may serve as a useful indicator for the early detection, risk stratification, and targeted therapy of patients with HNSC [11]. We used the CellMiner™ database to study a potential correlation analysis between drug sensitivity and *HPRT1* expression in pan-cancer, and the results showed that *HPRT1* expression was positively correlated with drug sensitivity to chelerythrine, fenretinide, ifosfamide, and obatoclax. Several studies have confirmed that chelerythrine has a wide range of antitumor activities. In PRAD, chelerythrine regulates the matrix metalloproteinase/matrix metalloproteinase/nuclear factor-κB system and inhibits the proliferation and metastasis of cancer cells [27]. In addition, chelerythrine limits the progression of GBM by suppressing the transforming growth factor beta 1 (TGFB1)-ERK1/2/Smad2/3-Snail/zinc-finger E-box binding protein 1 (ZEB1) signaling pathway [40]. Fenretinide has some efficacy in treating lung cancer, BRCA, PRAD, BLCA, COAD, CESC, UCEC, and HNSC [41–43]. Ifosfamide is often used in combination therapy with other drugs and has been widely used in treating malignant tumors [44]. As a general inhibitor of antiapoptotic proteins in the BCL2 family, obatoclax can synergistically inhibit the development of LIHC in combination with anti-PD-1 antibodies and effectively inhibit THCA growth [45, 46].

In five tumors (COAD, HNSC, LUAD, THCA, and UCEC), we performed immunohistochemical analysis of HPRT1 in tumor and normal tissues. The experimental results proved that it was expressed at higher levels in tumor tissues. The positive expression is at the site of the active cell proliferation position, a possible link between HPRT1 and tumor tissue proliferation activity.

In the immunotherapy analysis of HNSC, the expression of the *HPRT1* gene showed heightened sensitivity to CTLA4 and PD-1 combination therapy. In addition, we explored HNSC ceRNA networks (LINC00894-hsa-miR-299-5p-HPRT1, SNHG1-hsa-miR-299-5p-HRT1, SNHG12-hsa-let-7c-5p-HPRT1, NEAT1-hsa-let-72c-5p-HPRT1, and MSC-AS1-hsa-miR-23b-5p-HPRT1), which, in turn, offer new directions for further understanding the *HPRT1’s* mechanisms.

The innovation of the article stems from two key aspects. First, other researchers in the past only analyzed individual tumors. In contrast, our study conducted a comprehensive pan-cancer analysis of *HPRT1*, unveiling previously unexplored insights into the expression, survival prognosis, and immune-related situations of tumor types. Second, regarding the analysis of *HPRT1* in HNSC, based on previous studies, we further explored the relationship between this gene and PD-1, revealing the inhibitory effect of immunotherapy on the expression of this gene.

However, limitations exist, such as a smaller sample size in immunohistochemistry and the need for larger sample sizes and deeper and more comprehensive pan-cancer HPRT1 analysis may provide insights for future exploration of the role of HPRT1 in cancer. The question of how HPRT1 interacts with identified sensitive drugs influencing tumor progression remains unclear. There are many tumors (such as THCA) whose relationship with HPRT1 has never been discovered before our study, and further exploration is needed by future scholars. In addition, the role of genes closely related to *HPRT1*, such as *GMPR2* and phosphoribosyl transferase domain containing (*PRTFDC1*), in pan-cancer needs to be addressed.

## Conclusion

In this study, the expression, clinical prognostic value, and potential mechanisms of HPRT1 in various cancers were preliminarily explored, revealing that this gene exists as a carcinogenic gene in various tumors and is closely related to the immune microenvironment of tumors. *HPRT1* emerges as a promising biomarker for predicting and treating diverse cancer types.

## Data Availability

The datasets presented in this study can be found in the online repository as follows: TCGA (https://xena.ucsc.edu/; https://portal.gdc.cancer.gov/), TCIA (https://tcia.at/), GEO (https://www.ncbi.nlm.nih.gov/geo/).
